# SERS Hotspot Engineering by Aerosol Self‐Assembly of Plasmonic Ag Nanoaggregates with Tunable Interparticle Distance

**DOI:** 10.1002/advs.202201133

**Published:** 2022-06-07

**Authors:** Haipeng Li, Padryk Merkl, Jens Sommertune, Thomas Thersleff, Georgios A. Sotiriou

**Affiliations:** ^1^ Department of Microbiology, Tumor and Cell Biology Karolinska Institutet Stockholm SE‐17177 Sweden; ^2^ RISE Research Institutes of Sweden Stockholm SE‐11486 Sweden; ^3^ Department of Materials and Environmental Chemistry Stockholm University Stockholm 10691 Sweden

**Keywords:** flame aerosol deposition, pesticide residue detection, plasmonic nanoparticles, surface‐enhanced Raman scattering (SERS), SERS substrate fabrication

## Abstract

Surface‐enhanced Raman scattering (SERS) is a powerful sensing technique. However, the employment of SERS sensors in practical applications is hindered by high fabrication costs from processes with limited scalability, poor batch‐to‐batch reproducibility, substrate stability, and uniformity. Here, highly scalable and reproducible flame aerosol technology is employed to rapidly self‐assemble uniform SERS sensing films. Plasmonic Ag nanoparticles are deposited on substrates as nanoaggregates with fine control of their interparticle distance. The interparticle distance is tuned by adding a dielectric spacer during nanoparticle synthesis that separates the individual Ag nanoparticles within each nanoaggregate. The dielectric spacer thickness dictates the plasmonic coupling extinction of the deposited nanoaggregates and finely tunes the Raman hotspots. By systematically studying the optical and morphological properties of the developed SERS surfaces, structure–performance relationships are established and the optimal hot‐spots occur for interparticle distance of 1 to 1.5 nm among the individual Ag nanoparticles, as also validated by computational modeling, are identified for the highest signal enhancement of a molecular Raman reporter. Finally, the superior stability and batch‐to‐batch reproducibility of the developed SERS sensors are demonstrated and their potential with a proof‐of‐concept practical application in food‐safety diagnostics for pesticide detection on fruit surfaces is explored.

## Graphical abstract

Robust surface‐enhanced Raman scattering (SERS) sensing surfaces are fabricated using one‐step flame nanoparticle deposition. The sensing surfaces exhibit superior stability and high batch‐to‐batch reproducibility, highlighting their potential in practical (bio)chemical sensing. The detection of pesticides on fruit surfaces demonstrates a proof‐of‐concept practical application in food safety diagnostics at the point of consumption.

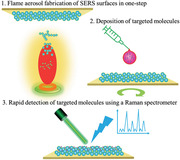

## 1 Introduction

Surface‐enhanced Raman scattering (SERS) is a powerful surface sensing technique that amplifies Raman scattering signals by analytes adsorbed onto metallic surfaces or nanostructured materials.^[^ ^1^, ^2^, ^3^ ^]^ Compared to traditional Raman spectroscopy, SERS has a high enhancement factor (EF) in the order of 10^8^–10^15^ and this high sensitivity promises the feasibility of single molecule detection.^[^ ^4^, ^5^, ^6^, ^7^, ^8^ ^]^ The phenomenon of SERS was first discovered in 1974 by Fleischmann et al., who reported the unusually intense Raman spectra of pyridine adsorbed on a roughened Ag (silver) electrode.^[^ ^9^ ^]^ In the past decades, SERS has undergone tremendous developments owing to its advantages of fingerprint recognition, high sensitivity, potential for point‐of‐care applications, and user‐friendliness.^[^ ^10^ ^]^ Therefore, this technique has been widely explored in the academic literature including but not limited to, agriculture, food, environmental monitoring, as well as chemical and biological sensing.^[^ ^2^, ^11^ ^–^ ^14^ ^]^

Chemical enhancement and electromagnetic enhancement are two proposed mechanisms to explain the amplified effect of SERS, with the latter mechanism considered to contribute more to the SERS enhancement than the former.^[^ ^10^, ^15^ ^]^ Chemical enhancement arises from the physicochemical interaction between analytes and the substrate, reaching an EF of 10^2^–10^4^.^[^ ^10^ ^]^ On the other hand, electromagnetic enhancement occurs by the local electromagnetic field on the metal nanoparticle surface through the effect of localized surface plasmon resonance.^[^ ^16^, ^17^, ^18^ ^]^ The electromagnetic radiation of incident light results in the collective oscillation of conduction band electrons at the interface of metallic structures (excitation of localized surface plasmons).^[^ ^1^, ^19^ ^]^ The local electromagnetic field is distributed nonuniformly and there are nanoscale regions (e.g., nanotips, interparticle nanogaps, and particle‐substrate nanogaps) with strong electromagnetic enhancements, the so‐called hotspots.^[^ ^20^ ^]^ Hotspots are desired to fabricate sensitive SERS sensing surfaces with high EF, because the molecules in hotspots contribute to most of the overall SERS signals (≈6% molecules contribute to ≈85% SERS signals^[^ ^21^ ^]^), as often calculated by measuring the signal from Raman reporters, such as the dye Rhodamine 6G (R6G).

However, although SERS exhibits unique advantages in (bio)chemical sensing, it is not broadly employed in practical applications. There are still important limitations on reproducible manufacturing of low‐cost SERS sensors while often the spatial non‐uniformity of the SERS surface results in unreliable measurements due to large variations in the sensor response—even within the same sensor.^[^ ^2^, ^21^, ^22^, ^23^ ^]^ Therefore, there is a need to employ and/or invent inexpensive SERS substrate nanomanufacturing processes that yield highly reproducible SERS sensing films with high uniformity. Among the plethora of SERS studies, most of them are based on plasmonic Ag or Au (gold) nanoparticles (NPs).^[^ ^24^, ^25^ ^]^ Although Ag exhibits lower plasmonic losses than Au,^[^ ^26^ ^]^ the usage of Ag NPs is limited because of their instability in aggressive environments, their oxidation with oxygen in atmospheric conditions, and their potential toxicity caused by the release of Ag ions by oxidative dissolution of their native oxide layer.^[^ ^27^, ^28^, ^29^, ^30^ ^]^ To overcome these limitations, coatings are often used to protect Ag NPs from degradation processes, and to introduce chemical, biological, and optical functionalities on their surface.^[^ ^31^, ^32^, ^33^, ^34^, ^35^, ^36^ ^]^

Here, Ag–SiO_2_ core–shell structured plasmonic nanoaggregates were produced by flame spray pyrolysis (FSP), a versatile, highly scalable and reproducible nanofabrication process.^[^ ^37^, ^38^, ^39^, ^40^ ^]^ The produced nanoparticles were in situ directly deposited on temperature‐controlled glass substrates through thermophoresis to fabricate the SERS sensing films. By controlling the amorphous SiO_2_ shell thickness, the hotspot formation and interparticle distance among the individual Ag NPs within the same nanoaggregate are finely tuned that in turn directly affect the NP plasmonic coupling and multimodal extinction. Even though spray flames yield highly reproducible NPs and deposited films, their potential in SERS sensing has not been extensively explored. Hu et al. have used FSP to prepare ultrathin SiO_2_‐coated Ag NPs giving rise to an EF of ≈10^5^ and a limit of detection (LOD) of 10^−10^ m for R6G.^[^ ^41^ ^]^ Fusco et al. have fabricated SERS films using direct gas‐phase deposition of Au nanoislands with tunable particle size and a high density of hotspots, resulting in a high EF of 10^6^–10^8^ for R6G.^[^ ^42^ ^]^ Despite these two promising results focusing on R6G molecules, the practical and reproducible application of flame aerosol deposited SERS surfaces has not been demonstrated, yet. To address this gap, here we first linked the SERS performance of flame aerosol deposited Ag–SiO_2_ core–shell nanoaggregate films to their hotspots generated by the controlled plasmonic coupling and multimodal extinction profiles. Then we investigated the long‐term stability and reproducibility of the developed SERS sensing NP films toward their practical application for detecting pesticide residues on fruit surfaces.

## 2 Results and Discussions

### 2.1 SERS Sensing Films Fabrication by Flame Aerosol Particle Synthesis and Deposition

**Figure 1:**
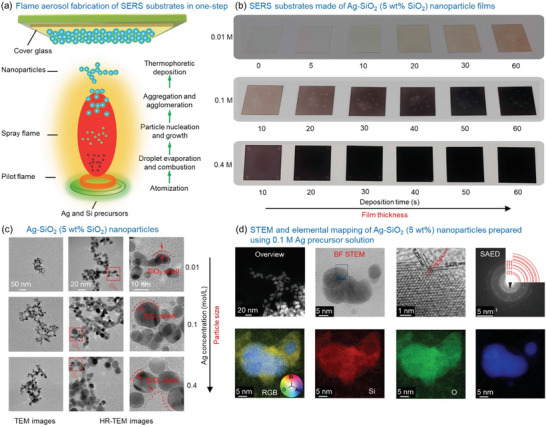
a) Schematic diagram of the experimental procedure to fabricate SERS sensing films in one‐step. b) Three groups of SERS films prepared using the precursor solutions with Ag concentrations of 0.01, 0.1, and 0.4 m. The nominal SiO_2_ content in the final Ag–SiO_2_ NPs was 5 wt%. c) TEM and high‐resolution (HR) TEM images of Ag–SiO_2_ NPs prepared using the precursor as above. The SiO_2_ shell is highlighted using red dashed lines in the third column. The TEM images in each column have the same scale bar. d) STEM and EDX mappings of Ag–SiO_2_ NPs prepared using 0.01 m Ag precursor solution and with SiO_2_ content of 5 wt%. In (d), the first row (from left to right) shows a wide field‐of‐view of fractal NPs, a high resolution BF STEM image of representative NPs, an enlargement from a region marked in the BF STEM image (denoted with a blue square), and a selected area electron diffraction (SAED) pattern from a large collection of particles. Lattice planes corresponding to spacings of 0.236 nm (Ag 111) and 0.204 nm (Ag 200) have been additionally marked in panel 3, indicating that this nanoparticle is oriented along the [011] zone axis. The SAED image is also overlaid with a simulated ring diffraction pattern from Ag nanoparticles (red rings in the SAED image), and the lowest order indices are provided. The second row exhibits EDX/EELS fusion images from the same region with false colors showing the elemental compositions of silicon (red), oxygen (green), and silver (blue), as well as their composite image (RGB).

Flame aerosol deposition was employed to rapidly fabricate SERS films in one‐step (**Figure** **1**a). The solution containing Ag and Si precursors was fed to a capillary and then atomized by pure oxygen into small droplets. These fine droplets were ignited by a pilot flame to form a spray flame, where nanoparticles were generated through droplet evaporation and combustion, particle nucleation, growth by coalescence and sintering, aggregation, and agglomeration.^[^ ^37^ ^]^ The freshly formed NPs were directly deposited on a temperature‐controlled glass substrate by thermophoresis. During flame nanoparticle synthesis, the particle size can be controlled by adjusting precursor concentrations,^[^ ^43^ ^]^ liquid precursor feed rate, and dispersion oxygen rate,^[^ ^44^ ^]^ while the film thickness can be tuned by adjusting the deposition time as well as the distance between the flame and the substrate.^[^ ^45^ ^]^

Three groups of SERS films were fabricated by depositing Ag–SiO_2_ NPs using precursor solutions with Ag concentrations of 0.01 m, 0.1 m, and 0.4 m for deposition times *t*_d_ = 10–60 s (Figure 1b). The flame aerosol deposition is a rapid (within seconds) process that allows for homogeneous film fabrication on large areas (e.g., up to dm^2^ in lab settings) essentially diminishing the barrier of high‐costs that are typically associated with the fabrication of SERS surfaces by other processes (e.g., lithography). The characteristic yellow plasmonic Ag color on the SERS films appears in Figure 1b for low deposition times that progressively becomes darker for increasing deposition times due to the thicker particle films (Figure S1, Supporting Information). Furthermore, increasing Ag concentration in the precursor solution increases the primary Ag particle size as shown in the transmission electron microscopy (TEM) images in Figure 1c (particle size distributions in Figure S2a–e, Supporting Information). Higher Ag precursor concentration results in more nucleation seeds in the flame and in higher coagulation rates with increased sintering rates of primary particles.^[^ ^46^ ^]^ The characteristic fractal morphologies of the plasmonic Ag–SiO_2_ nanoaggregates are shown in both the TEM (Figure 1c) and the scanning transmission electron microscopy (STEM) images (Figure 1d, upper left image). These images verify the formation of the Ag–SiO_2_ (5 wt% nominal SiO_2_‐content) core–shell morphology of the nanoaggregates in which the individual Ag spherical particles are separated by an amorphous SiO_2_ layer (see high‐resolution TEM (HR‐TEM) images in Figure 1c).^[^ ^33^ ^]^ The TEM images here are primarily used to identify the morphology of the nanoparticles dispersed in suspension, however, the Raman tests are performed on deposited films.

The crystallinity of the Ag NPs can be seen in the BF (bright field) STEM image (Figure 1d, the second and third panels), Selected area electron diffraction (SAED, shown in panel 4 of Figure 1d), and can also be verified by X‐ray diffraction (Figure S3). The formation of core–shell morphology was further investigated and confirmed with an elemental mapping technique known as hypermodal data fusion^[^ ^33^, ^47^, ^48^ ^]^ that combines coregistered electron energy‐loss spectroscopy (EELS) and energy‐dispersive X‐ray spectroscopy (EDX) datasets (Figure 1d, the second row images). These maps show that the same crystalline Ag NPs from panels 2 and 3 are surrounded by an amorphous shell of Si and O. The SiO_2_ shell exhibits a thickness of 1–4 nm. It should be noted that hermetically coated Ag nanoparticles with a nanothin amorphous SiO_2_ layer may be achieved with the employment of an enclosed flame reactor.^[^ ^31^, ^32^ ^]^ However, in this study the target of the SiO_2_ was to control the Ag interparticle distance instead of fully encapsulating the core Ag nanoparticles, so the open flame reactor was employed. It should also be noted that there are 9 or 4 small holes in the deposition holder (Figure S4a,b, Supporting Information), which are designed to attach the glass substrates with vacuum. The temperature of these holes is higher than the other areas of the deposition holder during flame spray deposition (Figure S4c, Supporting Information) that causes lower cooling of the contacted glass substrate parts and further results in less deposition of NPs (Figure S4, Supporting Information). However, these less‐deposited spots were avoided in later SERS measurements.

**Figure 2:**
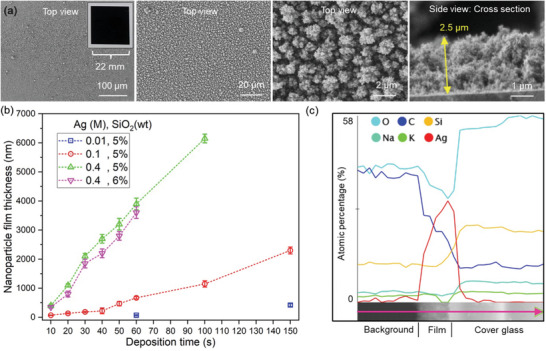
a) SEM images of a representative SERS sensing film from top view and side view. The SERS substrate was prepared using 0.1 m Ag precursor solution, nominal 5 wt% SiO_2_ content, and deposition time *t*_d_ = 150 s. The SERS glass substrate has a size of 22 mm * 22 mm as shown in the inset in the first SEM image. The deposited nanostructured film has a thickness of 2.5 µm, as shown in the cross‐sectional SEM image. b) Film thickness of SERS films prepared using 0.01, 0.1, and 0.4 m Ag precursor solutions with 5 wt% SiO_2_, as well as using 0.4 m Ag precursor solution with 6 wt% SiO_2_ content. c) A line EDX measurement (pink horizontal arrow in the rotated −90° SEM image) across background, nanoparticle film and glass substrate. The SERS film was prepared using 0.4 m Ag precursor solution, 6 wt% SiO_2_, and the deposition time *t*_d_ = 40 s. All data are represented mean ± standard deviation of the mean (SD) (*n* = 3).

The fabricated nanoparticle films are uniform at a large scale (**Figure** **2**a, see more images in Figure S5, Supporting Information) with a high porosity of 97% (Table S1, Supporting Information, for the calculation of nanoparticle film porosity).^[^ ^49^, ^50^ ^]^ The film thickness increases with longer deposition times independent of precursor molarity or SiO_2_ content (Figure 2b).^[^ ^51^ ^]^ The elemental composition of a line in a cross‐sectional scanning electron microscopy (SEM) image of an SERS film in Figure 2c shows the variations in atomic percentages of oxygen, carbon, silicon, sodium, potassium, and silver, identifying the presence of the nanoparticle film on the glass substrate.

**Figure 3:**
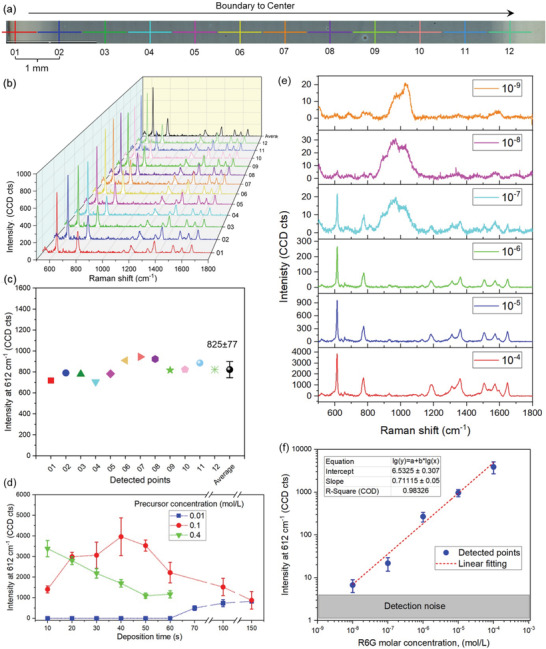
SERS performance evaluation with Raman confocal microscopy (with SERS film made using 0.1 m Ag solution, 5 wt% SiO_2_, and deposition time *d*_t_ = 150 s). a) 12 points on the SERS film detected b) by Raman microscopy and their corresponding SERS spectra. The Raman bands of 612, 773, 1190, 1361, and 1651 cm^−1^ are assigned to the vibrations of C–C–C ring in‐plane bending, C–H out‐of‐plane bending, C–O–C stretching, aromatic C–C stretching, and aromatic C–C stretching, respectively.^[^ ^54^, ^55^ ^]^ c) The intensity values of 612 cm^−1^ peaks and their average value. d) SERS performance evaluation on three groups of SERS films as a function of deposition time. e) Limit of detection (LOD) on the SERS sensing film (made using 0.1 m Ag precursor solution, 5 wt% SiO_2_, and *t*_d_ = 40 s). SERS spectra from the detection of R6G molecules dissolving into ethanol at the concentrations of 10^−4^ to 10^−9^ m (from bottom to top). f) Log–log plot of the averaged peak intensities at the Raman shift of 612 cm^−1^ as a function of the R6G molar concentration. All data are represented mean ± SD (*n* = 6–12).

SERS measurements on these three groups of sensing films (Figure 1b) were performed using confocal Raman microscopy. To study the feasibility of these SERS films, 10^−4^ m R6G in ethanol was spin‐coated on them and then detected by a 532 nm laser. 12 detection points were selected from the boundary to the center of the substrate with the distance between two successive points of 1 mm (**Figure** **3**a, with an SERS film made by a 0.1 m Ag precursor solution, 5 wt% SiO_2_, and the deposition time *t*_d_ = 150 s). The SERS spectra from all 12 detection points are similar and all of them show the typical Raman bands of R6G molecule (Figure 3b). The highest intensity peak appears at 612 cm^−1^, and thus this value is used to assess the SERS performance as sensor response *S*_R_. Furthermore, the average sensor response *S*_R_ of these 12 detected points exhibits a relative standard deviation of <10% (9.3%) highlighting the high spatial uniformity of the deposited SERS film. Upon examining the R6G intensity from all produced SERS films, the one prepared using 0.1 m Ag precursor solution and the deposition time *t*_d_ = 40 s shows the highest *S*_R_ (Figure 3d). Therefore, this SERS substrate was selected for further experiments to examine the reproducibility, LOD, and EF. Upon dissolving R6G molecules into ethanol at increasing concentrations (range 10^−9^ to 10^−4^ m) and measuring their SERS spectra (Figure 3e), typical Raman bands of R6G were observed down to 10^−9^ m, indicating an LOD of 10^−8^ m for this analyte (Figure S6, Supporting Information, shows the Raman spectra of the glass substrate in the presence and absence of the NP film).^[^ ^52^ ^]^ Upon logarithmic plotting the *S*_R_ as a function of the R6G molar concentration, a linear relationship was shown (Figure 3f) revealing a calculated LOD and LOQ (limit of quantitation)^[^ ^53^ ^]^ of 0.86*10^−8^ and 2.88*10^−8^ m, respectively.

From these spectra, an EF on the order of 10^6^–10^8^ was calculated using R6G as the analyte (Table S2 and Figure S7, Supporting Information). The SERS performance and EF are highly dependent on the adsorption of the analyte on the substrate surface. Lower LOD and higher EF are obtained for substrates immersed in R6G solution for a longer time, that increases the actual surface concentration of molecules.^[^ ^56^, ^57^ ^]^ For example, the LOD of porous Ag SERS substrates increases from 10^−8^ to 10^−13^ m when the R6G adsorption time increases from a few minutes to 8 h.^[^ ^58^ ^]^ In our SERS sensing surfaces, even though there is a short adsorption time of 2.5 min, their SERS performance and EF reach competitive values (Table S3, Supporting Information). The above results were also reproduced using a portable Raman spectrometer instead of a confocal Raman microscope (Figure S8, Supporting Information). The portable Raman spectrometer has a larger spot diameter (190 µm vs 3 µm) and a lower energy density (1.76*10^4^ mW cm^−2^ vs 7.96*10^7^ mW cm^−2^) than the Raman confocal microscopy system (Table S3, Supporting Information), offering advantages for in‐field and on‐site measurements. It should be noted that the SERS performance is linked to the plasmonic particle size. For example, by tuning the Ag–SiO_2_ (5 wt% SiO_2_) nanoparticle production process conditions here (i.e., increasing precursor feed rate and decreasing dispersion oxygen rate) results in increased average Ag crystal size from 8, 12, 15 to 19 nm (Figure S9a,b, Supporting Information) by promoting particle nucleation number and prolonging high‐temperature particle residence time.^[^ ^59^, ^60^ ^]^ The SERS performance of the fabricated substrates is almost linearly enhanced with the increasing nanoparticle size (Figure S9c,d, Supporting Information). Enlarging particle size enhances the electromagnetic field surrounding the particle, and thus increases the SERS enhancement.^[^ ^61^, ^62^ ^]^

### 2.2 Fabrication of SERS Films with High Sensitivity and Stable Performance

**Figure 4:**
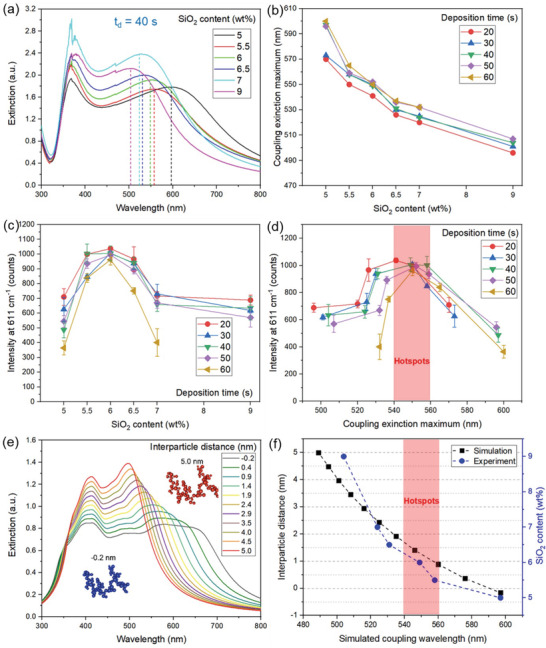
a) UV–vis spectra of 6 SERS films for various SiO_2_ contents (5–9 wt%, Ag = 0.4 m and *t*_d_ = 40 s). b) Coupling extinction maximum wavelengths *λ*_max_ of SERS films for various *t*_d_ (SiO_2_ contents 5–9 wt%). c) The average intensity at 611 cm^−1^ as a function of SiO_2_ contents in Ag–SiO_2_ NPs for various *t*_d_. d) The average intensity at 611 cm^−1^ as a function of coupling extinction maximum wavelengths *λ*_max_. e) Simulated extinction spectra of fractal‐like nanoparticle agglomerates with increasing interparticle distances (−0.2 to 5.0 nm, from top to bottom). Two simulated nanoaggregates with interparticle distances of −0.2 and 5.0 nm are also shown. f) The comparison of coupling extinction maximum wavelengths *λ*_max_ between simulation and experiment. The shaded red area shows the wavelength range and interparticle distance for the hot‐spot formation. All data are represented mean ± SD (*n* = 4–6).

To further understand the fundamental properties that dictate the SERS performance, we produced Ag–SiO_2_ NP films varying their SiO_2_ content (5 to 9 wt%, see Figure S10, Supporting Information, for longer range 1.3 to 25 wt% SiO_2_, Figure S11, Supporting Information, for the TEM images, and Figure S12, Supporting Information, for STEM images and elemental mapping), and thus tuning their plasmonic coupling extinction.^[^ ^33^ ^]^ **Figure** **4**a shows the extinction spectra of the deposited Ag–SiO_2_ (*t*_d_ = 40 s) films for varying SiO_2_ contents, in which the single mode plasmonic peak of Ag nanospheres appears at ≈390 nm^[^ ^63^ ^]^ as well as their coupling extinction bands that largely extend to the near‐IR for all SiO_2_ contents (the coupling extinction maximum wavelengths *λ*_max_ are shown with dotted lines). Upon plotting the plasmonic coupling extinction *λ*_max_ as a function of SiO_2_ content (Figure 4b), the *λ*_max_ values monotonously decrease for increasing SiO_2_ contents, in agreement with the literature.^[^ ^33^ ^]^ Now, the R6G SERS sensor response *S*_R_ of these films (acquired by the portable Raman spectrometer) for various deposition times *t*_d_ = 20–60 s as a function of the SiO_2_ contents is shown in Figure 4c. Interestingly, for all deposition times *t*_d_, the maximum *S*_R_ is achieved for the films with the same SiO_2_ content of 6 wt% (the coupling extinction maximum *λ*_max_ remains similar for all deposition times, see Figure S13, Supporting Information), in good agreement with similarly made Ag/SiO_2_ nanoparticles.^[^ ^41^ ^]^ After the deposition of R6G molecules, the coupling extinction *λ*_max_ increases (red shift) because of the enhanced refractive index of the surrounding environment (Figure S14c, Supporting Information). In plasmonic structures, the SERS signal is strongest when the plasmonic coupling extinction *λ*_max_ is near the excitation wavelength and/or falls within the window between the excitation wavelength and the Raman‐shifted wavelength.^[^ ^1^, ^64^ ^]^ Indeed, plotting the sensor response *S*_R_ as a function of the plasmonic coupling extinction *λ*_max_ for all SERS sensing films produced here as shown in Figure 4d, reveals that there is an optimal coupling extinction *λ*_max_ = 540–560 nm independent of film thickness or SiO_2_ content. It should be noted that lower deposition time *t*_d_ = 10 s yields to thinner NP films resulting in partially coated glass substrates and yielding weaker plasmonic coupling phenomena. Nonetheless, even for the partially coated films of *t*_d_ = 10 s, the SERS performance is comparable to the fully coated glass substrates (Figure S15, Supporting Information).

To further validate the plasmonic coupling extinction of the produced SERS sensing films here for varying SiO_2_ contents and thus Ag interparticle distances within the same nanoaggregates, we performed simulations of the extinction spectra of similar Ag fractal‐like (fractal dimension 1.8, generated by FracVal, see the Experimental Section for more details) nanoaggregates (Figure 4e) in which small changes of a few nanometers in interparticle spacing were sufficient to shift the coupling plasmonic band several nanometers. These simulations allowed for a direct comparison of the coupling extinction *λ*_max_ between the simulated interparticle distance and the experimental nominal SiO_2_ contents (Figure 4f). Very little change to the extinction spectra were observed when the interparticle distances were kept constant and only the size distributions of the primary particles were changed, while applying the measured mean particle size and standard deviation from TEM images was also not sufficient to recreate this behavior (Figure S16, Supporting Information). Therefore, the SERS performance of the Ag–SiO_2_ films here directly correlates to the SiO_2_ content and plasmonic coupling extinction *λ*_max_ indicating that the optimal interparticle distance among the Ag nanoparticles resulting in strong hot‐spot formation is between 1 and 1.5 nm (red shaded area in Figure 4f), in good agreement with the 1 nm gap observed by DNA‐functionalized plasmonic nanoparticles.^[^ ^65^ ^]^

**Figure 5:**
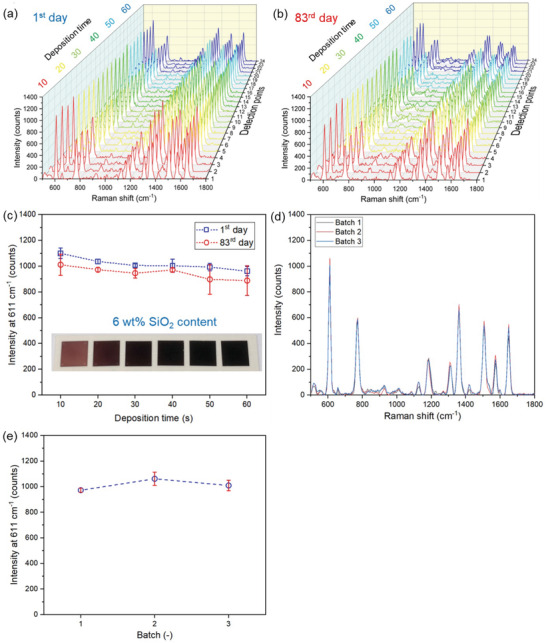
Stability and batch‐to‐batch reproducibility tests of SERS sensing films (6 wt% SiO_2_, Ag concentration of 0.4 m and *t*_d_ = 40 s). Two SERS measurements were performed on the same group of samples on a) 1st day (a) and b) on the 83rd day. For each sample, four points from different locations were measured. c) The average intensity at 611 cm^−1^ from the two SERS measurements. The inserted photo showing the SERS substrates with various deposition times (film thicknesses). d) The SERS tests on three independently made batches. These Raman spectra are averaged values from five or six different measurement points from the films. e) The average intensities at 611 cm^−1^ and standard deviations for the three batches in (d). All data are represented mean ± SD (*n* = 4–6).

To evaluate the stability and shelf‐life of the produced SERS sensing films here, we examined their performance over time. **Figure** **5** shows a collection of four spectra for each deposition time *t*_d_ immediately after their fabrication (a) and after 83 days (b). Upon averaging the *S*_R_ of these spectra and plotting them as a function of deposition time *t*_d_ (Figure 5c), there is a rather good agreement (3.2% to 9.6% difference) between the freshly made and the aged SERS films. This high stability of the produced Ag–SiO_2_ films here is attributed to the presence of the nanothin amorphous SiO_2_ coating that protects the core Ag NPs from oxidation and deterioration. Furthermore, we examined the batch‐to‐batch reproducibility of the produced SERS films here by fabricating three individual samples independently made at identical conditions. Their SERS spectra are largely overlapping (Figure 5d) while their average *S*_R_ obtains similar values (Figure 5e) highlighting the high reproducibility potential of flame aerosol deposited SERS sensing films (similar reproducibility was observed also using confocal Raman microscopy, see Figure S17, Supporting Information).

### 2.3 Rapid Detection of Pesticide Residues on Fruit Surface

**Figure 6:**
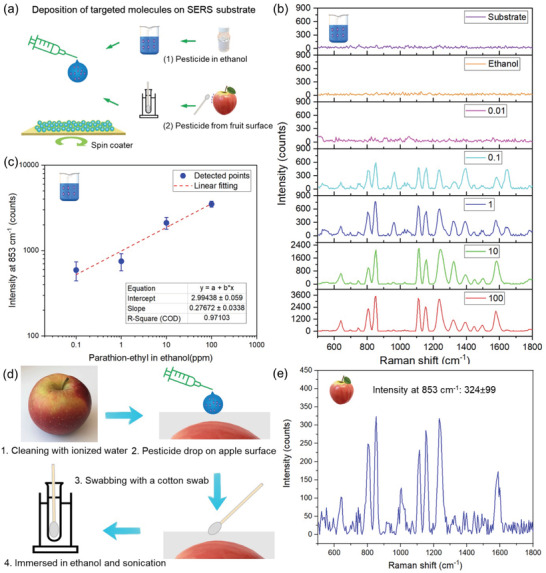
Rapid detection of pesticide residues in ethanol and on fruit surface. a) Deposition of targeted molecules on SERS film. Two kinds of solutions were tested: 1) parathion‐ethyl in ethanol, and 2) pesticide residues on apple surface. b) Raman spectra of parathion‐ethyl in ethanol for increasing from 0.01 to 100 ppm. Raman spectra of the SERS film alone (purple curve) as well as ethanol on the SERS films (orange curve) are provided as reference (average values from >5 measurements). c) Log–log plot of the average intensities at 853 cm^−1^ as a function of the parathion‐ethyl concentration. d) The operation process to detect pesticide residues on apple surface. e) The average SERS spectra from ten points from the detection of pesticide residues on apple surface. All data are represented mean ± SD (*n* = 10).

To examine the feasibility of the developed SERS sensing films in a realistic practical application, we detected the presence of a pesticide, parathion‐ethyl. We verified that parathion‐ethyl may be detected by SERS by first dissolving the pesticide in ethanol at the desired molar concentrations and subsequently spin‐coating the solutions on the SERS films (made with 0.4 m Ag precursor concentration, 6 wt% SiO_2_, and *t*_d_ = 40 s) (**Figure** **6**a). The resulting SERS spectra for increasing parathion‐ethyl concentrations (0.01–100 ppm) are shown in Figure 6b. The highest intensity is obtained at 853 cm^−1^ and upon plotting the intensities at this Raman shift as a function of the parathion‐ethyl concentration, the calibration curve can be formed detecting concentrations down to 0.1 ppm (Figure 6c) with LOD and LOQ of 0.04 and 0.15 ppm, respectively. As a proof of concept, a known concentration (2 mL of 100 ppm) of parathion‐ethyl was then placed on the surface (≈1 cm^2^) of a precleaned apple fruit (Figure 6a). Then, the pesticide residues were collected by a cotton swab that was subsequently immersed and sonicated in an ethanol solution (100 mL) to dissolve the pesticide molecules. Afterward, 2 µL of the solution was dropped on the SERS substrate for further SERS measurements on ten random spots on the substrates (process shown in schematic in Figure 6d). Figure 6e shows the average SERS spectrum of this nondestructive swabbing method that clearly shows the characteristic SERS peak of parathion‐ethyl, confirming its presence on the apple surface (Figure S18, Supporting Information, for control spectrum of the clean apple surface). Therefore, the fabricated SERS sensing films can rapidly detect pesticide residues on fruit surface for food safety diagnostics.

## 3 Conclusion

Here, we developed robust SERS sensing surfaces by flame aerosol deposition that combines particle synthesis and facile film fabrication in a cost‐effective and single process step. We demonstrate for the first time the aerosol deposition of flame‐made Ag–SiO_2_ nanoparticles on selected substrates toward their employment as highly uniform and reproducible SERS sensing surfaces. We identify the optimal morphology and structure to enhance the SERS hotspots, and highlight their performance not only with a Raman reporter but also in a practical application for the detection of pesticides on fruit surfaces. We focus on the synthesis of plasmonic Ag nanoaggregates and their rapid direct deposition on glass substrates that requires only seconds. The interparticle distance of the Ag nanoparticles within each nanoaggregate is finely tuned by the addition of amorphous SiO_2_ during the nanoparticle flame synthesis. This amorphous SiO_2_ is present as a nanothin coating surrounding the Ag nanospheres within the nanoaggregates acting as a dielectric spacer. The controlled interparticle distance by the SiO_2_ dielectric spacer directly affects the plasmonic coupling among the Ag nanoparticles generating hotspots with high signal enhancement potential. The structure–performance relationships are systematically studied and deciphered here revealing that the optimal interparticle distance among the Ag nanoparticles for the highest SERS sensor response of R6G from the generation of hotspots lies between 1 and 1.5 nm. The flame aerosol deposited Ag–SiO_2_ SERS sensing films exhibit also superior stability over time (shelf life >2.5 months) as well as high batch‐to‐batch reproducibility, highlighting the potential of SERS films made by this nanomanufacture process to be employed in practical (bio)chemical sensing. As a proof‐of‐concept for a realistic practical application, we detected the presence of pesticide on the surface of fresh fruits, indicating that such SERS films may be useful for food safety sensing at the point of consumption.

## 4 Experimental Section

### Chemicals

Silver acetate (99%, Alfa Aesar) and tetraethyl orthosilicate (98%, Sigma‐Aldrich) were used as the precursors for Ag and Si, respectively. The mixture of 2‐ethylhexanoic acid (99%, Sigma‐Aldrich) and acetonitrile (≥99.5%, Sigma‐Aldrich) with a volume ratio of 1:1 was used as the solvent. Ag precursor was first dissolved into the solvent under reflux at the temperature of 110 °C, and then Si precursor was added to get the desired silica mass fraction in the final produced Ag–SiO_2_ NPs.

### Flame Aerosol Fabrication of Glass‐Based SERS Substrates

Plasmonic nanoparticle films were synthesized and deposited on temperature‐controlled glass substrates using FSP.^[^ ^45^, ^66^ ^]^ The precursor solution was fed through a capillary (Kel F 7750‐12, HAMILTON) at a liquid feed rate by a syringe pump (New Era Pump Systems, Inc.) and then atomized into fine droplets by oxygen (>99.5%, Strandmöllen AB) at a gas flow rate of 5 mL min^−1^. A pilot flame was used to ignite the dispersed droplets and to maintain the spray flame. This pilot flame was premixed by methane (>99.5%, AGA Gas AB) and oxygen (>99.5%, Strandmöllen AB) at gas flow rates of 1.5 and 3.2 L min^−1^, respectively. A deposition holder was fixed above the nozzle at 22 cm. The colorless and clear cover glass (22 mm × 22 mm, 0.16–0.19 mm in thickness, Borosilicate glass D 263 M of the hydrolytic class 1 630–2760, VWR) was attached on the bottom of the deposition holder using a vacuum pump. The temperature of the deposition holder was controlled as 16 °C using a water bath (CORIO CD‐200F, JULABO). The NPs produced from the spray flame were directly deposited on the glass substrate through thermophoresis. The deposition time was tuned by using a metal shield plate, which blocked the glass substrate before and after deposition.

### Nanoparticle and Film Characterizations

TEM images of NPs were collected using a 120 kV LaB_6_ electron microscope (Talos 120C G2) with a Ceta‐D detector. During sample preparation, 1 mg produced NPs were dispersed into 2 mL ethanol. After sonication of around 5 min, two drops of the solution were placed on carbon films coated copper grid (S160‐4, Agar Scientific) and dried in the room temperature.

HR‐STEM and the elemental mapping were acquired on an aberration‐corrected transmission electron microscope (Thermo Fisher Scientific Themis Z, 300kV operation voltage) equipped with a Super‐X EDX detector (Thermo Fisher) and Quantum Gatan Image Filter (Gatan Inc.) operating in dual‐EELS mode. The elemental maps were extracted from a predictive model of the EDX dataset that was generated by using spectral variations from the coregistered core‐loss and low‐loss hyperspectral EELS datasets. In this case, the dispersion of the EELS spectrometer was configured to capture Ag‐*N*, Ag‐*M*, Si‐*L*, C‐*K*, and O‐*K* edges while simultaneously recording full EDX spectra at each probe position during a scan of the region of interest. The variations in these EELS edges were used to track compositional variations and provided a chemically justifiable means for in‐painting the EDX data, which were much sparser due to the short exposure time (4.5 ms) and lower collection efficiency. The EDX maps from Figure 1d as well as Figure S13 of the Supporting Information were generated using the hypermodal data fusion method by simultaneously combining low‐loss EELS, core‐loss EELS, and EDX datasets via weighted matrix concatenation. Weights were assigned by first normalizing all datasets by their total variance and then additionally reducing the magnitude of the EDX weighting by a factor of 1000. Technical details for the method are outlined in Thersleff et al.^[^ ^47^, ^48^ ^]^ and an additional demonstrative example from these materials are provided in Section S7 of the Supporting Information.

SEM images of nanoparticle films (Figure 3a; Figure S5, Supporting Information) were collected using a field emission scanning electron microscope (JSM‐7401F, JEOL) incorporating a cold cathode field emission gun.

The nanoparticle film thickness was measured using a desktop SEM with an FEG source (Thermo Scientific Phenom Pharos). The SERS substrate was cracked into pieces. The piece with a vertical cross‐section was attached on a 90° angles SEM pin stub (10‐002206‐10, Micro to Nano) using a conductive double sided adhesive carbon tab (15‐000412, Micro to Nano). An EDX sensor was equipped for elemental analysis. The collected SEM images were analyzed using the software ImageJ to obtain the film thickness values at three different places for each sample. One representative example is shown in Figure S1 of the Supporting Information.

X‐ray diffraction (XRD) measurements were done using a compact desktop powder X‐ray diffractometer (Rigaku MiniFlex) in the 2*θ* angle range of 10°–80° with a step width of 0.01° and the speed of 1° min^−1^. The diffraction data analysis was conducted using PDXL2 software to get crystal sizes of particles.

UV–vis measurements were performed using a spectrophotometer (Analytik Jena Specord 210 Plus) in the range of 300–1200 nm with the step width of 1 nm, the integration time of 0.1 s and the speed of 10 nm s^−1^.

### The Deposition of Target Molecules on SERS Substrates

Rhodamine 6G (83697, Sigma‐Aldrich) was used as the Raman reporter to assess the performance of SERS substrates. R6G molecules were dissolved into ethanol at the concentrations of 10^−4^, 10^−5^, 10^−6^, 10^−7^, 10^−8^, and 10^−9^ m. One of pesticide chemicals, parathion‐ethyl (≥99.6%, 45607, Sigma‐Aldrich), was dissolved into ethanol at volume fractions of 100, 10, 1, 0.1, and 0.01 ppm, which correspond to 4.65 × 10^−4^, 4.65 × 10^−5^, 4.65 × 10^−6^, and 4.65 × 10^−7^ m, respectively.

Spin‐coating was used to deposit target molecules on the surface of SERS substrates. The spin coater (Laurell Technologies WS‐650Mz) rotated the attached SERS substrate at an initial spin speed of 100 rpm for 30 s, and during this period 150 µL solution was placed on the substrate surface. Then, the spin speed was accelerated to 2000 rpm for 2 min to deposit uniform molecule films by the centrifugal force.

### SERS Measurements

The SERS measurements on R6G molecules were done using both a confocal Raman microscopy system and a portable Raman spectrometer system. The Raman microscopy system contains a confocal Raman imaging microscopy (Alpha 300RAS, Witec GmbH) equipped with UHTS 300 VIS spectrometer and a laser with the excitation wavelength of 532 nm. During SERS measurements, the laser power of 1 mW, the integration time to 0.5 s per spectrum, the number of accumulations of 10, the objective (Zeiss EC Epiplan 20×/0.4), the grating of G2:1800 g mm^−1^ BLZ = 500 nm were set to obtain Raman spectra from the Raman shift range of 500–1800 cm^−1^. The used 20× objective resulted in a spot size of about 3 µm in diameter and an analysis depth of ≈500 nm. For each sample, a certain number of spots were characterized, covering a line from the edge of the substrate to the center. The presented spectra are the average spectra of these single spectra. All raw data were analyzed in the Witec Project Plus 5.1 software, correcting for cosmic rays and removing the background.

The Raman spectrometer system was bought from Ocean Insight, which composes of the 532 nm laser with adjustable output power, Raman coupled fiber probe, Preconfigured QEPRO for 532 nm Raman, and Raman SERS substrate Holder 9.5 mm. The laser spot has a diameter of 190 µm and a depth of field of ≈2.2 mm. The OceanView 2.0 spectroscopy software was used for data acquisition and analysis. The SERS measurements on R6G molecules were performed using the laser power of 2 mW, the integration time of 0.5 s and the scans of three times. The SERS measurements on parathion‐ethyl molecules were done using the laser power of 20 mW, the integration time of 5 s, and the scans of two times.

The LOD and LOQ were also calculated using the signal‐to‐noise method, which is commonly applied to analytical method that exhibit baseline noise

(1) $$\mathrm{LOD}=\frac{3n}{S/D}$$

(2) $$\mathrm{LOQ}=\frac{10n}{S/D}$$

where *n* and *S*/*D* are the concentration of the analyte and signal‐to‐noise ratio, respectively.

To facilitate the actual application, the fabricated SERS substrate was used to rapidly detect pesticide residues on apple skin. The apple surface was contaminated with parathion‐ethyl and then the residues were collected using a nondestructive swabbing method (Figure 6d). The operation process is described below: the apple was bought in local supermarket and cleaned twice with ionized water. Then, 2 µL 100 ppm parathion‐ethyl in ethanol solution was deposited on the apple surface (≈1 cm^2^) and dried at room temperature. A cotton swab was prewetted with ethanol and swabbed across the apple surface for 60 s. The swab was immersed in 100 µL ethanol and then sonicated for 2 min. 2 µL of the solution was dropped on the SERS substrate. After drying in room temperature, the SERS test was done on ten random points.

### Simulation of the Plasmonic Extinction Spectra

The plasmonic extinction spectra were simulated by generating fractal‐like polydisperse aggregates using FracVal^[^ ^67^ ^]^ and applying the coupled dipole approximation implemented by Auguié^[^ ^68^ ^]^ as described previously.^[^ ^33^ ^]^ Both simulation codes are available for free in the links: https://github.com/padmer/FracVAL_cda_helpers ^[^ ^33^ ^]^ and http://nano‐optics.ac.nz/cda/.^[^ ^68^ ^]^ In order to better represent the overall extinction spectra the mean of 30 different aggregates was calculated. The parameters used to generate the aggregates were: 100 primary particles, fractal dimension of 1.8, fractal prefactor of 1.3, geometric mean of 12 nm unless otherwise specified and geometric standard deviation of 1.2 unless otherwise specified.

### Statistical Analysis

Preprocessing of data, analyzed number (*n*) of samples and repeat times are listed for each experiment as described above. Quantitative data are presented as mean ± standard deviation of the mean (SD) as indicated in the figure legends. All statistical analyses of Raman data, XRD data, TEM particle size distribution data, SEM film thickness data, and UV–vis data were performed using the Origin software. All schematic diagrams were made using the Microsoft PowerPoint software.

## Conflict of Interest

The authors declare no conflict of interest.

## Author Contributions

H.L. was involved in conceptualization, nanoparticle synthesis and film deposition, data curation, data collection, formal analysis, methodology, visualization, and writing and editing the original draft. P.M. was involved in conceptualization, setup trainings, formal analysis, result discussions, suggestions, methodology, and plasmonic interparticle coupling simulation. J.S. was involved in Raman microscopy measurements and related result discussions. T.T. was involved in EDX mapping as well as related visualization, methodology, and writing. G.A.S. was involved in conceptualization, supervision, formal analysis, funding acquisition, methodology, resources, visualization, and writing and editing the original draft. All authors reviewed the results and approved the final version of the manuscript.

## Supporting information

Supporting Information

## Data Availability

The data that support the findings of this study are available from the corresponding author upon reasonable request.
