# Supplementary material for: SERS Hotspot Engineering by Aerosol Self‐Assembly of Plasmonic Ag Nanoaggregates with Tunable Interparticle Distance
Source: Adv Sci (Weinh). 2022 Jun 7;9(22):2201133. doi: 10.1002/advs.202201133 (PMC9353460; doi:10.1002/advs.202201133)
Supplement: Supplementary file 1 — Supporting Information [file ADVS-9-2201133-s001.pdf]

Supporting Information

SERS Hotspot Engineering by Aerosol Self-Assembly of Plasmonic Ag Nanoaggregates with Tunable Interparticle Distance

*Haipeng Li, Padryk Merkl, Jens Sommertune, Thomas Thersleff, and Georgios A. Sotiriou**

H. Li, P. Merkl, G.A. Sotiriou
Department of Microbiology, Tumor and Cell Biology, Karolinska Institutet, SE-17177 Stockholm, Sweden.
E-mail: georgios.sotiriou@ki.se

J. Sommertune
RISE Research Institutes of Sweden, SE-11486 Stockholm, Sweden.

T. Thersleff
Stockholm University, Department of Materials and Environmental Chemistry, Stockholm 10691, Sweden

Table of Contents

Section S1: SERS sensing films fabrication by flame aerosol particle synthesis and deposition. 6

Figure S1. One representative example to show film thickness measurement using a desktop SEM instrument. 6

Figure S2. Particle size distributions of Ag-SiO_2 NPs used in this work. 7

Figure S3. The XRD signals of the fabricated SERS substrates used in this work. 8

Figure S4. The two deposition holders used in this work. (a) The deposition holder with 9 vacuum holes, and its one representative fabricated SERS substrate. (b) The deposition holder with 4 vacuum holes, and its one representative fabricated SERS substrate. (c) The temperature distributions of the cover glass during flame spray deposition. 9

Figure S5. SEM images (Top view) of two representative SERS substrates prepared using 0.01 M Ag precursor solution (a) and (c), as well as using 0.1 M Ag precursor solution (b) and (d). 10

Table S1. Calculation of porosity of nanoparticle films deposited on SERS substrates. The SERS substrates were deposited by Ag-SiO_2 NPs (6 wt% content of SiO_2) using 0.4 M Ag precursor solution. 11

Figure S6. The Raman spectra from the cover glass and the SERS substrate without R6G deposition. 12

Section S2: The calculation of enhancement factor of the fabricated SERS substrate. 13

Table S2. Calculation of enhancement factor (EF) for R6G molecules deposited Ag-SiO_2 nanostructured SERS substrates. The tests were done using the Raman microscopy system. 14

Figure S7. (a) SERS and (b) normal Raman measurements on R6G molecules using the Raman microscopy. 15

Table S3. Comparison of SERS performance of silver micro/nano structured substrates using R6G as an analyte. 16

Section S3: The SERS measurements using a portable Raman spectrometer system. 17

Table S4. Comparison between the sophisticated Raman microscope system and the portable Raman spectrometer system used in this work 17

Figure S8. The SERS measurements using a portable Raman spectrometer system. 18

Section S4. SERS performance optimization by increasing the nanoparticle size. 20

Figure S9. SERS performance optimization by increasing the nanoparticle size. The substrates were prepared using 0.1 M Ag solution, 5 wt% SiO_2, and deposition time d_t = 40 s. (a) The two photos showing the FSP deposition process. The distance between the deposition holder and the flame top was maintained as 8-9 cm. The F/O (precursor feed rate and dispersion oxygen rate, mL/min and L/min) were varied from 5/5, 5/3, 8/3 to 12/3 to provide more precursors in the spray flame and to enlarge the flame size. (b) The XRD signals of the produced SERS substrates. The crystal sizes of nanoparticles increase from 8, 12, 15 to 19 nm when F/O were used as 5/5, 5/3, 8/3 and 12/3, respectively. (c) SERS spectra from the four groups of substrates using the Raman spectrometer. For each substrate, four points were detected. (d) The intensity values of 611 cm^{-1} peaks and their averaged value. All data are represented mean ± SD (n = 4). 20

Section S5. Fabrication of SERS films with high sensitivity and stable performance. 21

Figure S10. The intensity values at 611 cm^{-1} of SERS substrates using core-shell structured Ag-SiO_2 NPs with SiO_2 contents of 1.3 wt%, 2 wt%, 4 wt%, 6 wt%, and 25 wt%. 21

Figure S11. (a) Five groups of SERS substrates prepared using core-shell structured Ag-SiO_2 NPs with SiO_2 contents of 5.5 wt%, 6 wt%, 6.5 wt%, 7 wt%, and 9 wt%. 22

Figure S12. Ag-SiO_2 NPs prepared using 0.4 M Ag precursor solution and with the SiO_2 content of 6% in NPs. (a) STEM image showing the overview of fractal NPs. (b) High resolution BF STEM image of representative NPs and (c) their corresponding Selected Area Electron Diffraction (SAED) patterns. The diffraction rings in the SAED image fit well with the simulated results of Ag particles (red rings in the SAED image), indicating the presence of Ag NPs. (d) HAADF image of representative NPs. (e), (f), (g), (h), and (i) exhibit EDX/EELS fusion images with false colors

showing the elemental compositions of silicon (f), oxygen (g), carbon (h), and silver (i). The Red-Green-Blue (RGB) color model was used in (e). 23

Figure S13. UV-Vis spectra of SERS substrates before (solid curves) and after (dashed curves) R6G deposition. The SERS substrates were deposited by core-shell structured Ag-SiO_2 NPs with SiO_2 contents of 6 wt%........ 24

Figure S14. The first extinction peak positions before and after R6G deposition in UV-Vis spectra of SERS substrate (a) and their difference (b). The second extinction peak (LSPR λmax) positions before and after R6G deposition in UV-Vis spectra of SERS substrate (c) and their difference (d). 25

Figure S15. (a) The photo showing SERS substrates composing of core-shell structured Ag-SiO_2 NPs with SiO_2 contents of 6 wt%. The deposition times of 10 s, 20 s, 30 s, 40 s, 50 s, and 60 s were used to increase the film thickness. During FSP deposition, 0.4 M Ag precursor solution was used. The two SEM images (side view) of the substrates with deposition times of 10s and 20s were shown to indicate film thicknesses. (b) Coupling extinction maxima of two groups of SERS substrates with deposition times of 10 s and 20 s. These two groups of SERS substrates compose of core-shell structured Ag-SiO_2 NPs with SiO_2 contents of 5 wt%, 5.5 wt%, 6 wt%, 6.5 wt%, 7 wt%, and 9 wt%. During FSP deposition, 0.4 M Ag precursor solution was used. (c) The intensity values of 611 cm^{-1} peaks and their averaged values as a function of SiO_2 contents in Ag-SiO_2 NPs. (d) The intensity values of 611 cm^{-1} peaks and their averaged values as a function of coupling extinction maxima. 26

Figure S16. Simulation of extinction spectra of fractal like aggregates with (a) varying primary particle size and (b) with the primary particles sizes and geometric standard deviations corresponding to those measured from TEM images of aggregates synthesized with a given silica weight percentage (shown in Figure S2). PP in (a) represents primary particle. 27

Figure S17. The SERS tests on three batches using a confocal Raman microscopy. The SERS substrates were prepared using 0.1 M Ag precursor solution, 5 wt% SiO_2 and the deposition time

t_d = 40 s. These Raman spectra are averaged values from 6 different measurement points from the substrate. The averaged intensities of the detected points at 612 cm^{-1} are 3955 ± 916 CCD cts, 3951 ± 625 CCD cts, and 3868 ± 916 CCD cts, indicating good reproducibility of the fabrication process. 28

Section S6. Rapid detection of pesticide residues on fruit surface. 29

Figure S18. (a) The Raman spectra of 100 ppm parathion-ethyl in ethanol and its vibrational descriptions. The insert image shows the molecule structure of parathion-ethyl. The SERS spectra of parathion-ethyl exhibit peaks at 853 cm^{-1}, 1109 cm^{-1}, 1239 cm^{-1}, 1324 cm^{-1}, 1398 cm^{-1}, and 1579 cm^{-1}, which are assigned to the vibrations of P-O stretching, C-N stretching, C-O stretching, C-H bending, N-O stretching and phenyl stretching, respectively.[18] (b) The reference experiment for the rapid detection of pesticide residues on apple surface. During this reference experiment, pure ethanol instead of parathion-ethyl in ethanol was used and the rest operation process was the same as the pesticide experiment. 29

Section S7. The hypermodal data fusion method for EDX/EELS mapping 30

Figure S19. One representative example showcasing the benefits of the hypermodal data diffusion method for EDX/EELS mapping. (a) Raw EDX (after applying a spatial spectral blur to estimate missing values). (b) EDX after data fusion. Ag-SiO_2 NPs were prepared using 0.4 M Ag precursor solution and with the SiO_2 content of 6 wt% in NPs. The second, third, fourth, and fifth column exhibit images with false colors showing the elemental compositions of silicon, oxygen, carbon, and silver. The Red-Green-Blue (RGB) color model was used in first column. Note that, although the color yellow was used for the Carbon maps in column 4, this element was not included in the RGB composite image: the yellow here originates from mixing between Si (red) and O (green). The EDX mapping images have the same scale bar 30

References 31

Section S1: SERS sensing films fabrication by flame aerosol particle synthesis and deposition.

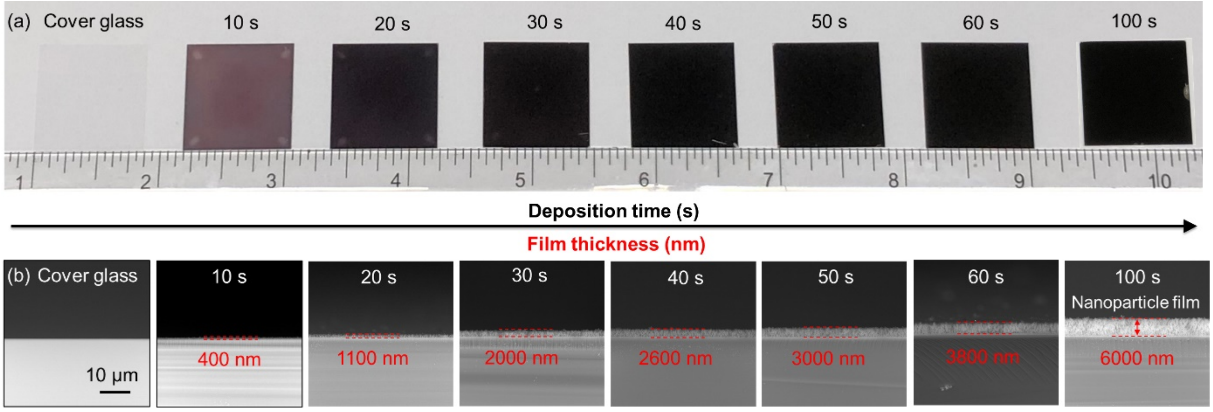

Figure S1. One representative example to show film thickness measurement using a desktop SEM instrument. The SERS substrates were prepared using 0.4 M Ag precursor solution to synthesize Ag-SiO_2 NPs (5 wt% SiO_2). (a) Photos of SERS substrates with increasing deposition times from 10 s, 20 s, 30 s, 40 s, 50 s, 60 s to 100 s. Cover glass was shown as a reference. The ruler shows the length in inches. (b) SEM images to show the cross section of each substrate. The SEM images have the same scale bar as shown in the first image.

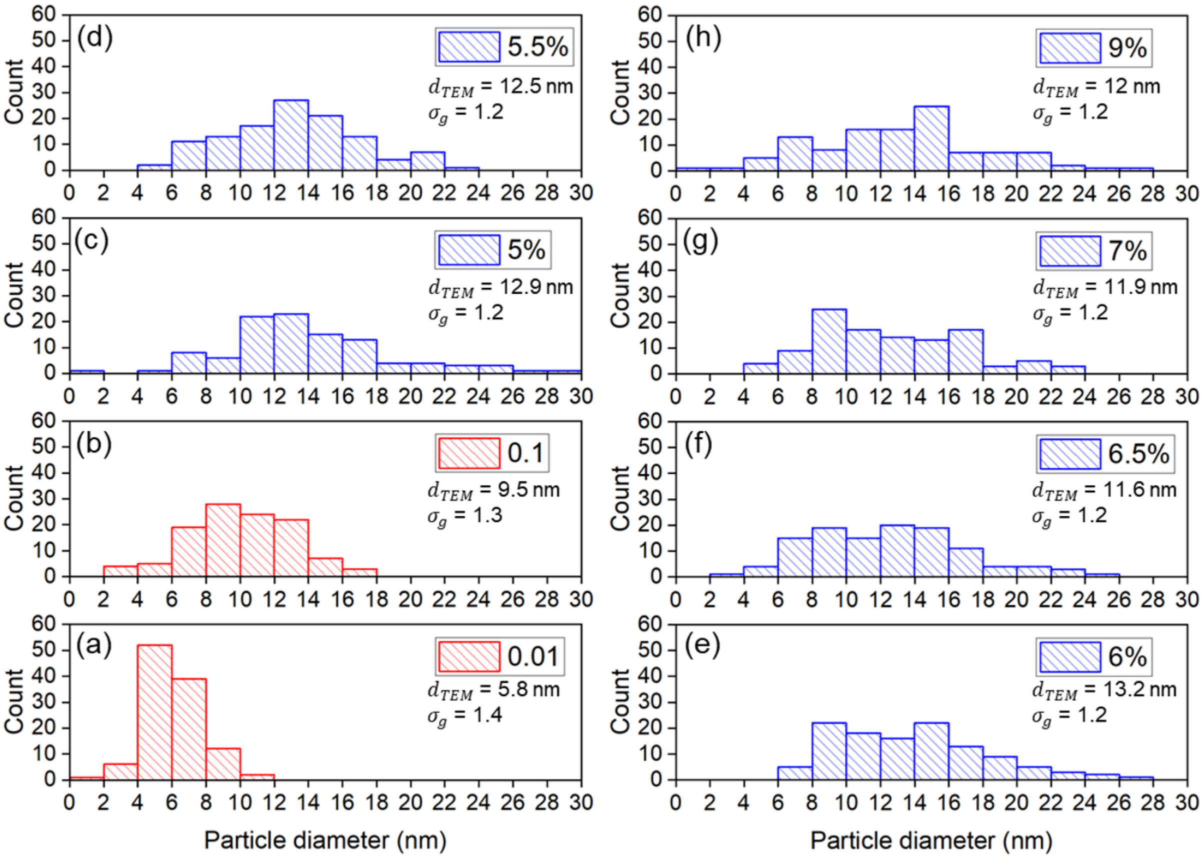

Figure S2. Particle size distributions of Ag-SiO_2 NPs used in this work. NPs with the SiO_2 content of 5 wt% were prepared using 0.01 M (a), 0.1 M (b) and 0.4 M (c) Ag precursor solutions. NPs with SiO_2 contents of 5.5 wt% (d), 6 wt% (e), 6.5 wt% (f), 7 wt% (g) and 9 wt% (h) were prepared using 0.4 M Ag precursor solutions. For each sample, more than 100 primary NPs were counted in high-resolution TEM images. The geometric mean particle size (d_{TEM}) and the geometric standard deviation (σ_g) of each sample are listed.

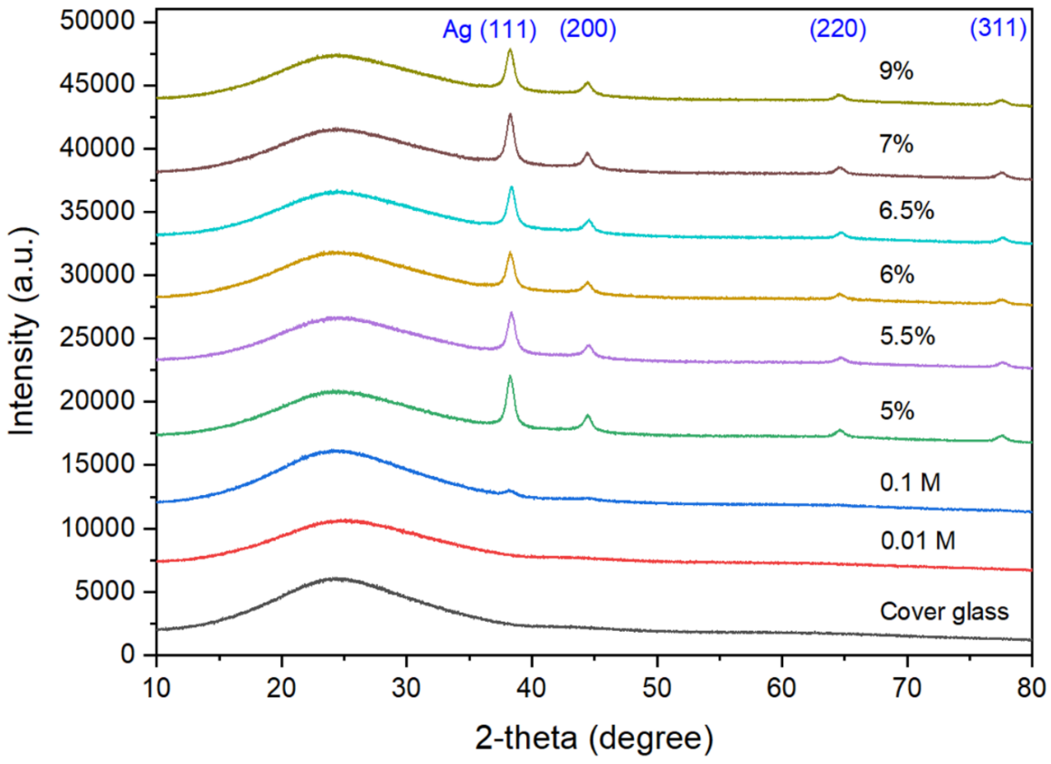

Figure S3. The XRD signals of the fabricated SERS substrates used in this work. From bottom to top: The XRD signal of cover glass was shown as a reference (black curve). The substrates were deposited by Ag-SiO_2 NPs with the SiO_2 content of 5 wt% using 0.01 M, 0.1 M, and 0.4 M Ag precursor solutions (red, blue, and green curves). NPs with using 0.1 M and 0.4 M Ag precursor solutions have crystal sizes of 8 nm and 12 nm, respectively. The rest five substrates were deposited by NPs with SiO_2 contents of 5.5 wt%, 6 wt%, 6.5 wt%, 7 wt%, and 9 wt% using 0.4 M Ag precursor solutions, which have crystal sizes of 12 nm, 12 nm, 12 nm, 12 nm, and 12 nm, respectively. The deposition times were not less than 50 s to ensure sufficiently thick films for XRD. The blue marks indicate the XRD peaks at 2-theta of 38.3°, 44.5°, 64.7°, and 77.7°, corresponding to (111), (200), (220), and (311) crystallographic planes of cubic silver crystals (COD 1509146, Space group F m -3 m). These peaks are barely obvious for the SERS substrate using 0.01 M Ag precursor solution due to the weak XRD signals diffracted from the very thin nanoparticle film (less than 50 nm, see Figure 1 b and Figure 2 b). The broad peak appearing at 2-theta of 24.2° is attributed to the borosilicate composition of the cover glass.

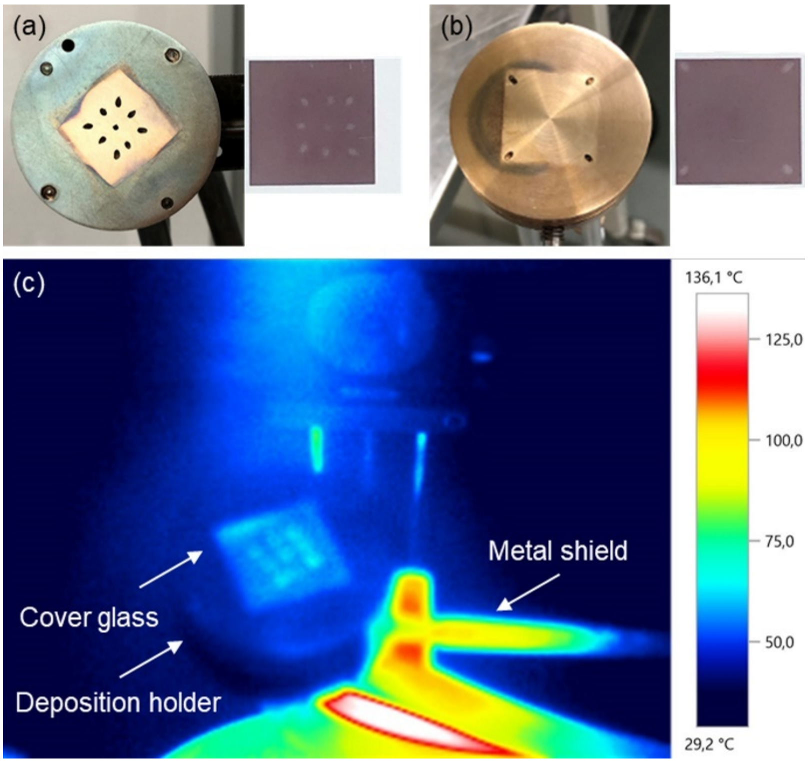

Figure S4. The two deposition holders used in this work. (a) The deposition holder with 9 vacuum holes, and its one representative fabricated SERS substrate. (b) The deposition holder with 4 vacuum holes, and its one representative fabricated SERS substrate. (c) The temperature distributions of the cover glass during flame spray deposition. The temperature was detected by an 871 thermal imaging camera (Testo). The temperature of the metal shield was high due to its near distance to the spray flame. The two SERS substrates were prepared using 0.1 M Ag precursor solution, 5 wt% SiO_2, and the deposition time t_d = 40 s. Compared to the first deposition holder, the second deposition holder was improved to distribute the less deposited spots near the substrate edges.

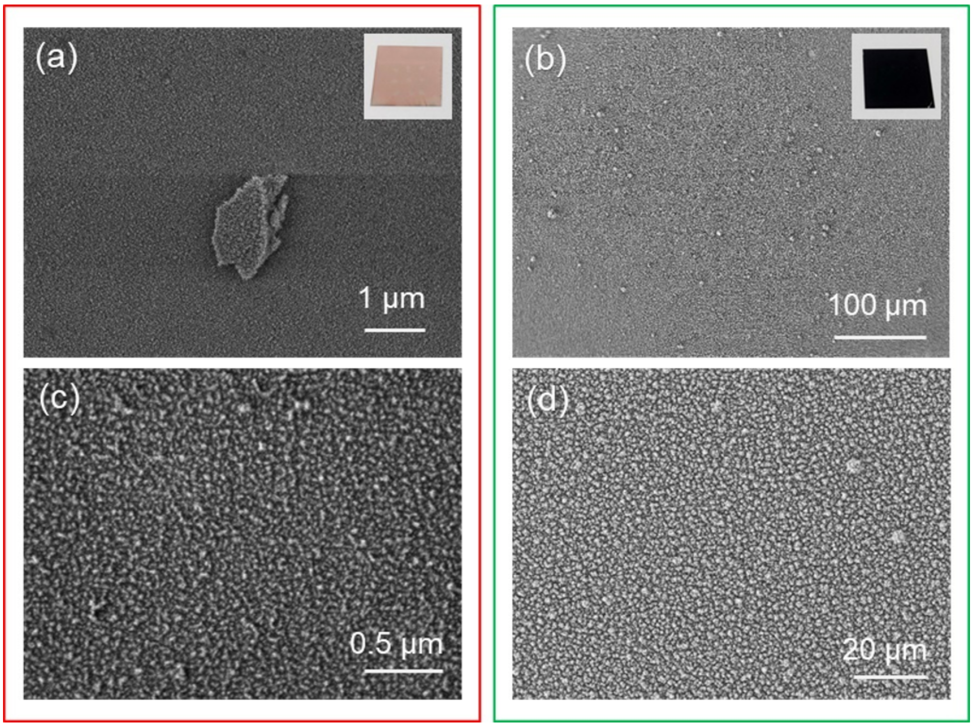

Figure S5. SEM images (Top view) of two representative SERS substrates prepared using 0.01 M Ag precursor solution (a) and (c), as well as using 0.1 M Ag precursor solution (b) and (d). The two inserted photos in (a) and (b) show SERS substrates (22 mm * 22 mm, Length * Width), which were prepared using in Ag-SiO_2 NPs (5 wt% SiO_2), and the deposition time t_d = 150 s. The fabricated nanoparticle films are uniform at a large scale. A contamination was shown in (a) which serves as a reference. The SEM images of (b) and (d) are shown in Figure 3 in the main text as well.

The nanoparticle film porosity was calculated using the equation:

$$\phi = 1 - \frac{m}{\rho A h} \quad (1)$$

where ϕ, m, ρ, A, and h are the film porosity, mass of the film, density of the particles, area of the film deposited on the cover glass, and film thickness, respectively. The mass of the film is obtained through weighing the cover glass before and after deposition. The density of particles was used as the mixed density of Ag and SiO_2, considering Ag-SiO_2 NPs has the SiO_2 content of 6 wt%. The area of the film was taken as the cover glass area. The film thickness was measured using SEM instruments.

Table S1. Calculation of porosity of nanoparticle films deposited on SERS substrates. The SERS substrates were deposited by Ag-SiO_2 NPs (6 wt% content of SiO_2) using 0.4 M Ag precursor solution.

Deposition time, t_d (s)	60	120
Film mass, m (mg)	0.55	1.08
Particle density, ρ (g/cm^3)	8.91	8.91
Area of film, A (cm^2)	4.84	4.84
Film thickness, h (nm)	3600	7800
Film volume, (cm^3)	$1.74*10^{-3}$	$3.77*10^{-3}$
Particle volume, (cm^3)	$6.17*10^{-5}$	$1.21*10^{-4}$
Film porosity, ϕ (-)	96.5%	96.8%

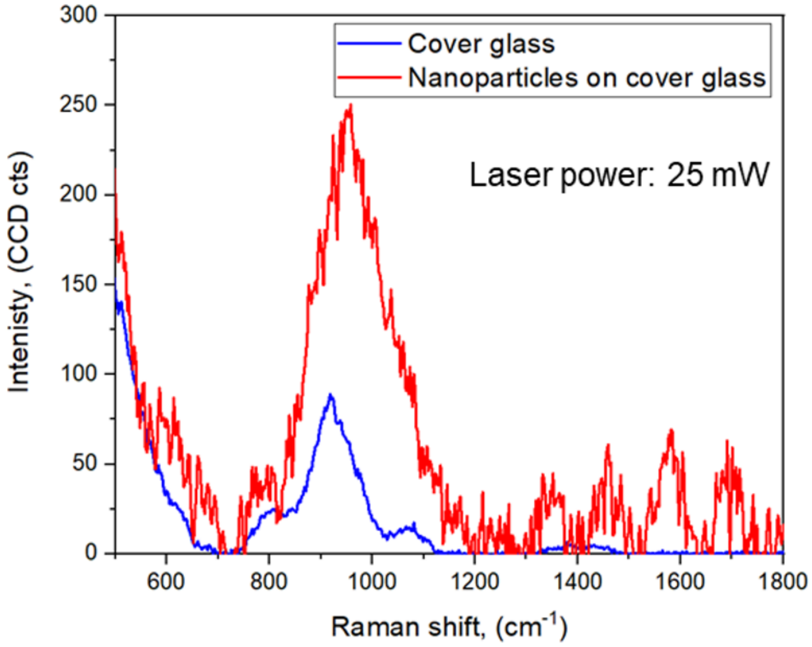

Figure S6. The Raman spectra from the cover glass and the SERS substrate without R6G deposition. The laser power was enhanced to 25 mW to obtain clear signals.

Section S2: The calculation of enhancement factor of the fabricated SERS substrate.

Enhancement factor (EF) is used to quantify the promoted Raman signal intensity per molecule by SERS compared to normal Raman. Here, EF is calculated using a hybrid approach, which is based on the measurements of normalized intensities and on the approximate estimation of the surface density of excited molecules. [1]

$$EF = \frac{[I_{SERS}/(P_{SERS}t_{SERS})]/n_{SERS}}{[I_{Normal}/(P_{Normal}t_{Normal})]/n_{Normal}} \tag{2}$$

where I, P, t, and n are the Raman intensity, laser power, accumulation time, and the number of effective molecules, respectively. The subscripts, $SERS$ and $Normal$, represent the measurements in the SERS test and the normal Raman test, respectively. The inputs and outputs of the calculation are listed in Table S2.

The SERS measurement was conducted on R6G molecules deposited on the Ag-SiO_2 nanostructured SERS substrate (Figure S7 a). 150 μL 10^{-4} M R6G in ethanol solution was first dropped on the surface of the SERS substrate, and then spin coating was done to form uniform R6G layer. The size of Ag-SiO_2 NPs is assumed as 8 nm, which is the same as the crystal size from the XRD measurement. Occupied Surface area of R6G is assumed as 1 nm^2 and R6G molecules are assumed to cover 2.3% of the totally surface of a nanoparticle.[2]

The normal Raman measurement was done on R6G powders (figure S7 b), which have the molecular weight of 479.01 g/mol and the density of 0.79 g/cm^3. The effective molecules are considered as the solid powders in the laser spot aera.

Table S2. Calculation of enhancement factor (EF) for R6G molecules deposited Ag-SiO_2 nanostructured SERS substrates. The tests were done using the Raman microscopy system.

	SERS		Normal Raman
Intensity, I (CCD cts) at 612 cm^{-1}	3955		13
Laser power, P (mW)	1		0.1
Accumulation time, t (s)	0.5		0.5
Laser spot area	3 μm spot radius 500 nm in depth		3 μm spot radius 500 nm in depth
Targeted sample	R6G molecules on the SERS substrate surface via spin coating		R6G powders
Effective molecule number, n (counts)	$1.0*10^3$	$1.5*10^5$	$3.5*10^9$
Assumption	Monolayer R6G and NPs	97% porosity	All molecules in the spot area
EF, (counts/mW/s)	$\sim1*10^8$	$\sim1*10^6$	1

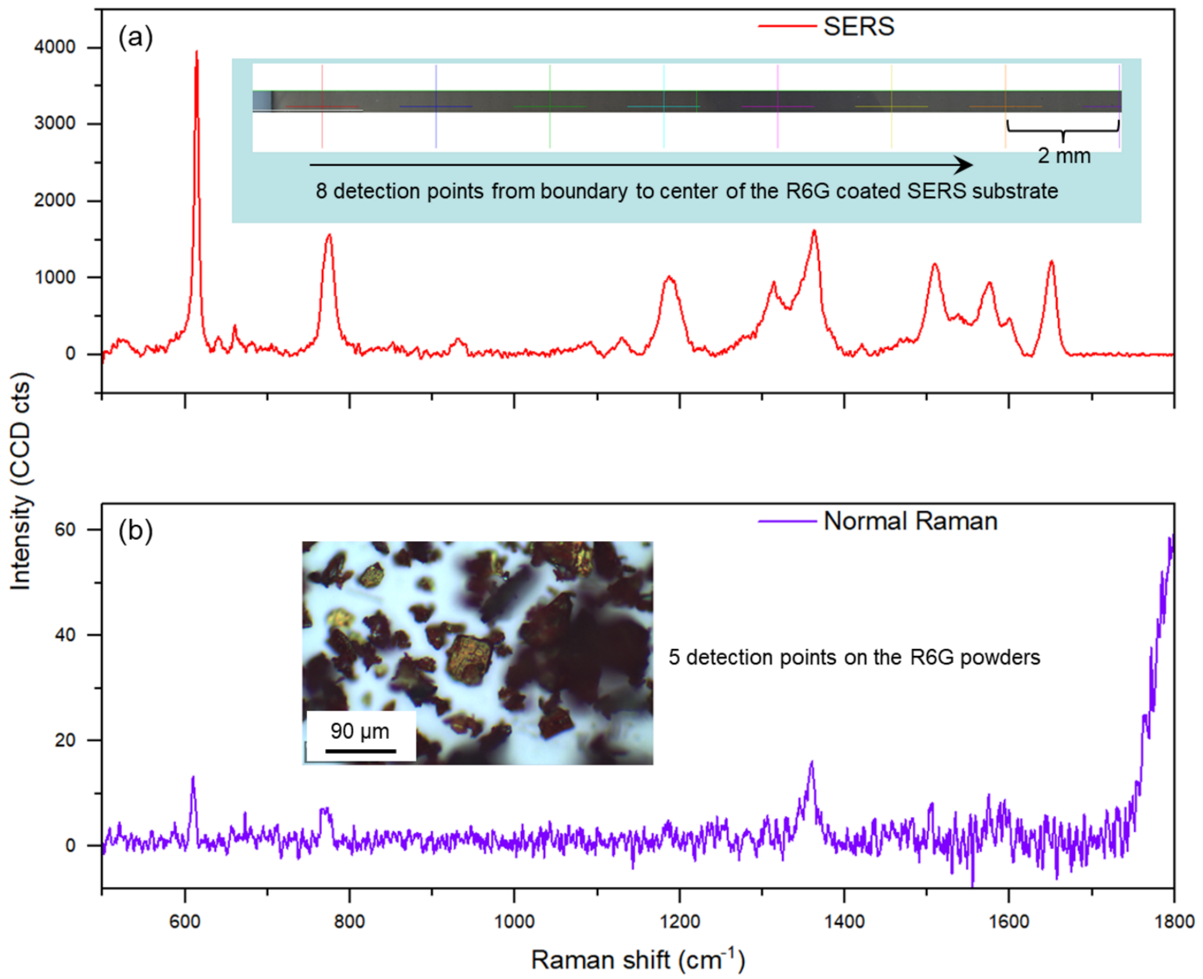

Figure S7. (a) SERS and (b) normal Raman measurements on R6G molecules using the Raman microscopy. The SERS substrate was prepared using 0.1 M Ag precursor solution, 5 wt% SiO_2, and the deposition time t_d = 40 s. The spectrum of R6G from SERS measurements is the averaged value of 8 detection points from the boundary to the center of the R6G coated SERS substrate (inserted photo in a). The spectrum of R6G from normal Raman measurements is the averaged value of 5 detection points form the R6G powders (inserted photo in b). The same laser with the wavelength of 532 nm was used for both the SERS and normal Raman measurements. While 1 mW was used for SERS measurement, the laser power was reduced to 0.1 mW for normal Raman measurement to avoid superheating powers. All data are represented mean ± SD (n = 6-8).

Table S3. Comparison of SERS performance of silver micro/nano structured substrates using R6G as an analyte. In each literature, only the optimal performance of the SERS substrate is cited.

Materials	Size[(a)] (nm)	Fabrication technique	λ, P, t[(b)]	Adsorption time[(c)]	LOD[(b)]	EF[(b)]	Ref.
Ag-SiO_2 NPs	8	Flame aerosol deposition	532, 1, 0.5	2.5 min	10^{-8}	10^{6}~10^{8}	This work
Ag NPs	29~58	Seed-mediated electroless deposition	785, 10, 10	30 min	/	10^{4}	[3]
Ag NPs	75	Sonication Centrifugation	785, 50, 0.5	5 min (Sonication)	<10^{-6}	10^{5}	[4]
Ag NPs	100~400	Sonochemistry	532, 2.3, 16	3 min	10^{-6}	/	[5]
Ag flowerlike	200~400	Electrodeposition	514, 20, 30	24 h	/	10^{7}	[6]
Ag nano dendrite	1200~3000	Electroless deposition	532, 25, 10	10 min	10^{-9}	10^{8}	[7]
Ag micro-flower	1300	Sonication	514.2, /, 1	24 h	10^{-17}	10^{14}	[8]
Ag NP@N-GQD[(d)]	85	Sonication Centrifugation	785, 50, 0.5	5 min (Sonication)	<10^{-6}	10^{6}	[4]
Ag NPs/Carbon	76	Flame deposition Magnetron sputtering	532, 0.7, 10	30 min	10^{-11}	10^{9}	[9]
Ag@CSs[(e)]	180	Hydrothermal method Solution method	514, 2, /	Few hours	10^{-10}	10^{6}	[10]
Ag_2O@Ag NPs	80	PVD Annealing	532, 48, /	/	10^{-11}	10^{7}	[11]
Ag/Si-NPAs[(f)]	35~55	Immersion-plating	532, /, 10	20 min	10^{-15}	/	[12]
MPA[(g)]/AgNPs	47	Roll-to-plate embossing Hydrothermal method	633, 0.1, 5	/	10^{-12}	10^{10}	[13]
Ag NPs/Si nanopillar	45	Droplet-confined electroless deposition	532, 7.92, 1	/	10^{-11}	/	[14]
Ag skeleton	710	Thermal induction	532, 3.6, 0.02	Few minutes	10^{-8}	10^{5}	[15]
				8 h	10^{-13}	/	
Ag dendrites-coated porous Si	100	Electrochemical anodic oxidation	532, 2, 1	2 h	10^{-11}	/	[16]

(a) Here the size means the averaged size or the size range of nano/micro structured material. (b) λ, P, t, LOD, and EF are excitation wavelength (nm), excitation power (mW), accumulation time (s), limit of detection (M), and enhancement factor, respectively. (c) Adsorption time means the time needed for the SERS substrate to absorb R6G molecules. The symbol of / means that the related information is not available. (d) Ag NP@N-GQD is silver NPs protected by small nitrogen-doped Graphene Quantum. (e) Ag@CSs represents core–shell silver–carbon nanocomposites. (f) Ag/Si-NPAs is silver/silicon nanoporous pillar arrays. (g) MPA means micropyramid array.

Section S3: The SERS measurements using a portable Raman spectrometer system.

Table S4. Comparison between the sophisticated Raman microscope system and the portable Raman spectrometer system used in this work. The excitation laser with a wavelength of 532 nm was used in both systems.

	Raman microscope	Raman Spectrometer
Laser power, P (mW)	1	2
Accumulation time, t (s)	0.5	0.5
Laser spot area	3 µm spot radius<br>500 nm in depth	190 µm spot radius<br>2.2 mm in depth
Surface area ratio	1	$9*10^{3}$
Surface energy density ratio	1	$2*10^{-4}$
Volume ratio	1	$4*10^{7}$
Volume energy density ratio	1	$5*10^{-8}$
Advantages	High detection resolution, precisely controlling detection locations, sophisticated	Facile, portable, user-friendly

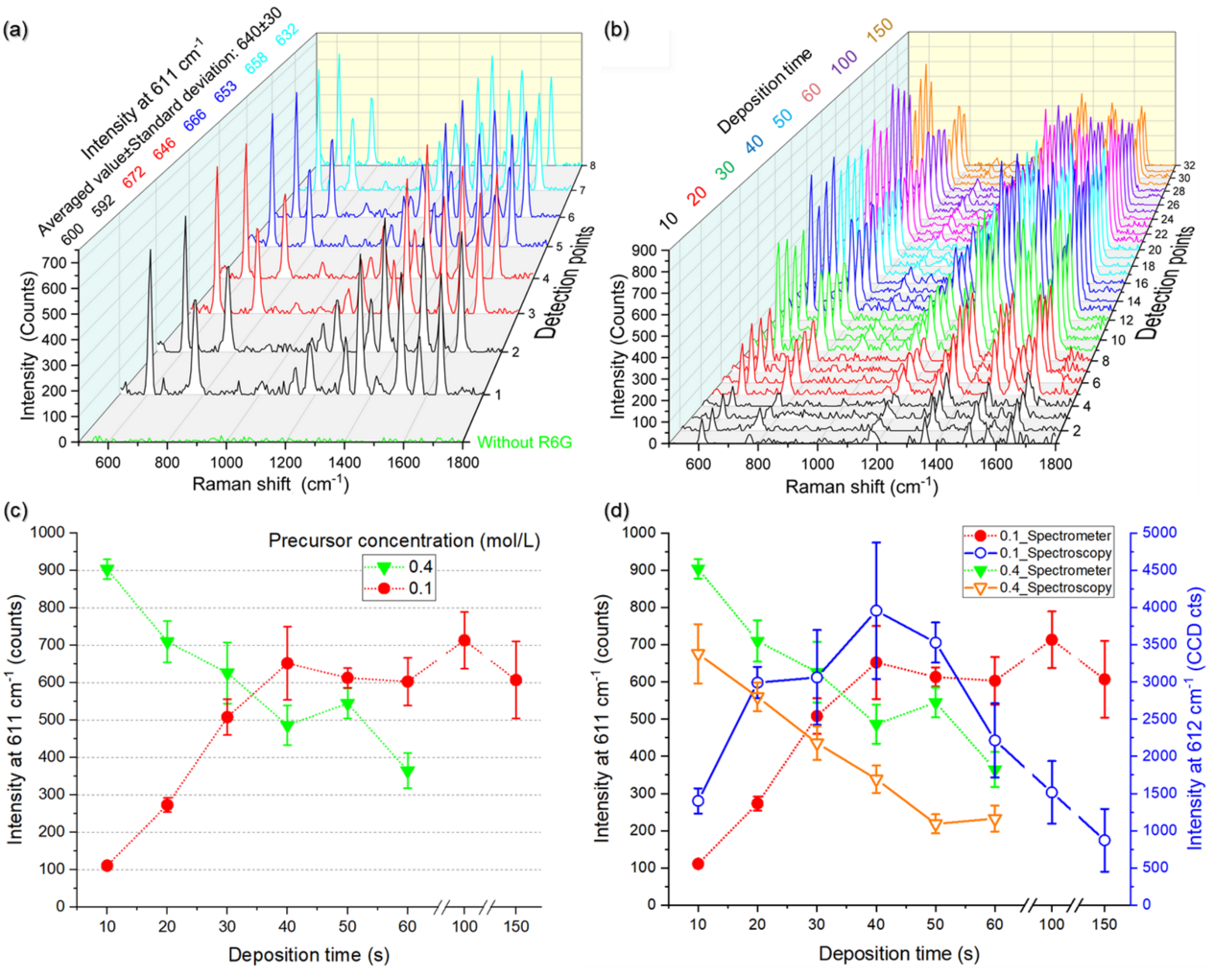

Figure S8. The SERS measurements using a portable Raman spectrometer system. (a) SERS spectra from one substrate using the Raman spectrometer system. This SERS substrate was prepared using 0.1 M Ag precursor solution and the deposition time t_d = 40 s. Before R6G spin coating, no clear Raman signal was observed (Green curve). After R6G spin coating, one point from the SERS substrate was detected 8 times. The interval between two consecutive times is 5 minutes. (b) SERS spectra from 8 substrates using the Raman spectrometer. For each substrate, four points were detected. These eight SERS substrates were prepared using 0.1 M Ag solution and the deposition times of 10 s, 20 s, 30 s, 40 s, 50 s, 60 s, 100 s and 150 s. The averaged intensities at 611 cm^{-1} and their standard deviations are shown in figure (c) (red points and error bars). (c) SERS performance evaluation on two groups of substrates using the Raman spectrometer system. These two groups of SERS substrates were prepared using Ag concentrations of 0.1 M and 0.4 M. (d) SERS performance comparison between the Raman microscopy system and the Raman spectrometer system. The similar trends were observed for SERS substrates using the Raman spectroscopy system and the Raman spectrometer system, excepted for the decreasing intensity from deposition time of 60 s, 100 s to 150 s (0.1 M). It is

caused by the small detection depth of the used laser beam, which could not detect all the effective molecules exposed at the substrate surface. All data are represented mean ± SD (n = 4-12).

Section S4. SERS performance optimization by increasing the nanoparticle size.

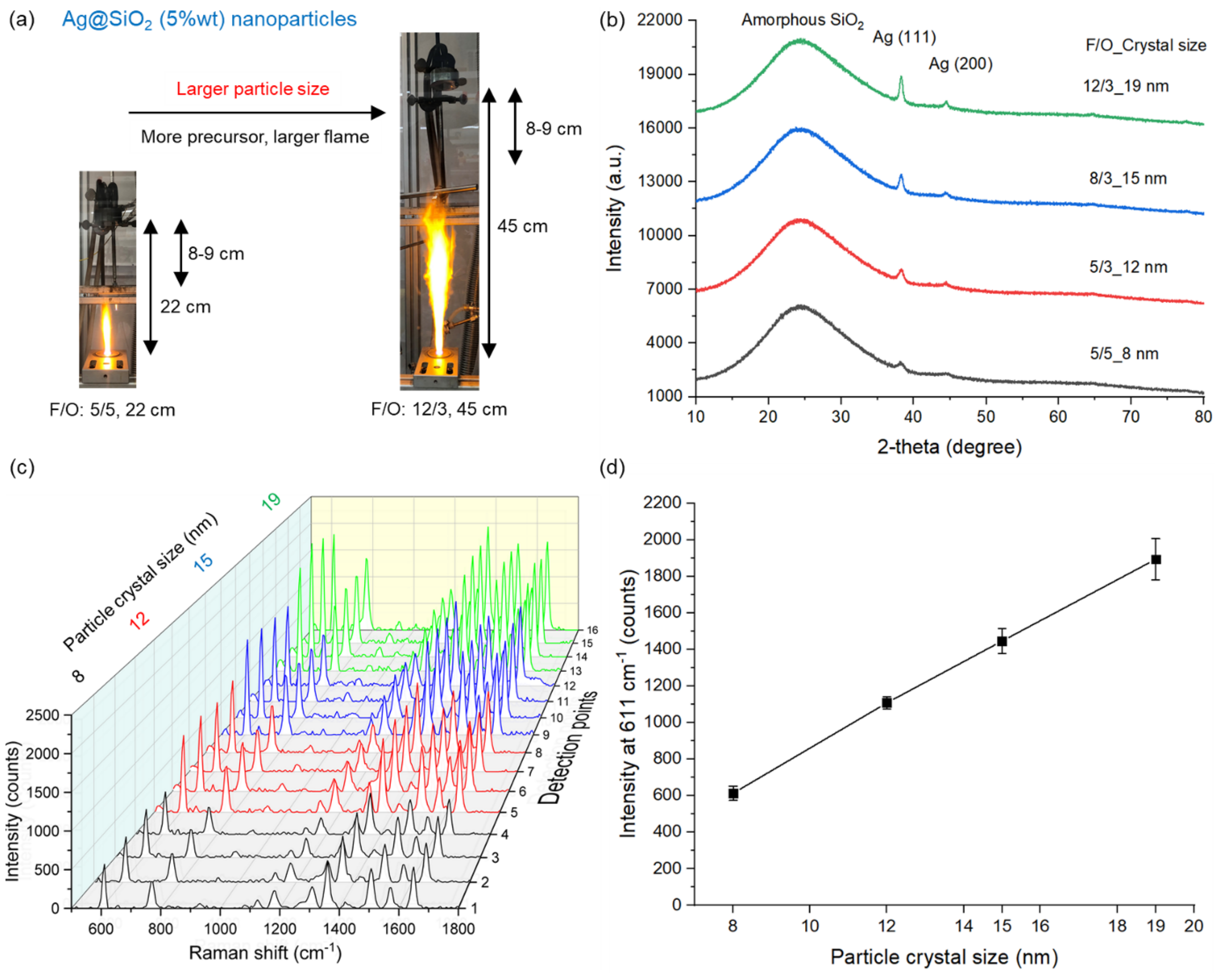

Figure S9. SERS performance optimization by increasing the nanoparticle size. The substrates were prepared using 0.1 M Ag solution, 5 wt% SiO_2, and deposition time d_t = 40 s. (a) The two photos showing the FSP deposition process. The distance between the deposition holder and the flame top was maintained as 8-9 cm. The F/O (precursor feed rate and dispersion oxygen rate, mL/min and L/min) were varied from 5/5, 5/3, 8/3 to 12/3 to provide more precursors in the spray flame and to enlarge the flame size. (b) The XRD signals of the produced SERS substrates. The crystal sizes of nanoparticles increase from 8, 12, 15 to 19 nm when F/O were used as 5/5, 5/3, 8/3 and 12/3, respectively. (c) SERS spectra from the four groups of substrates using the Raman spectrometer. For each substrate, four points were detected. (d) The intensity values of 611 cm^{-1} peaks and their averaged value. All data are represented mean ± SD (n = 4).

Section S5. Fabrication of SERS films with high sensitivity and stable performance.

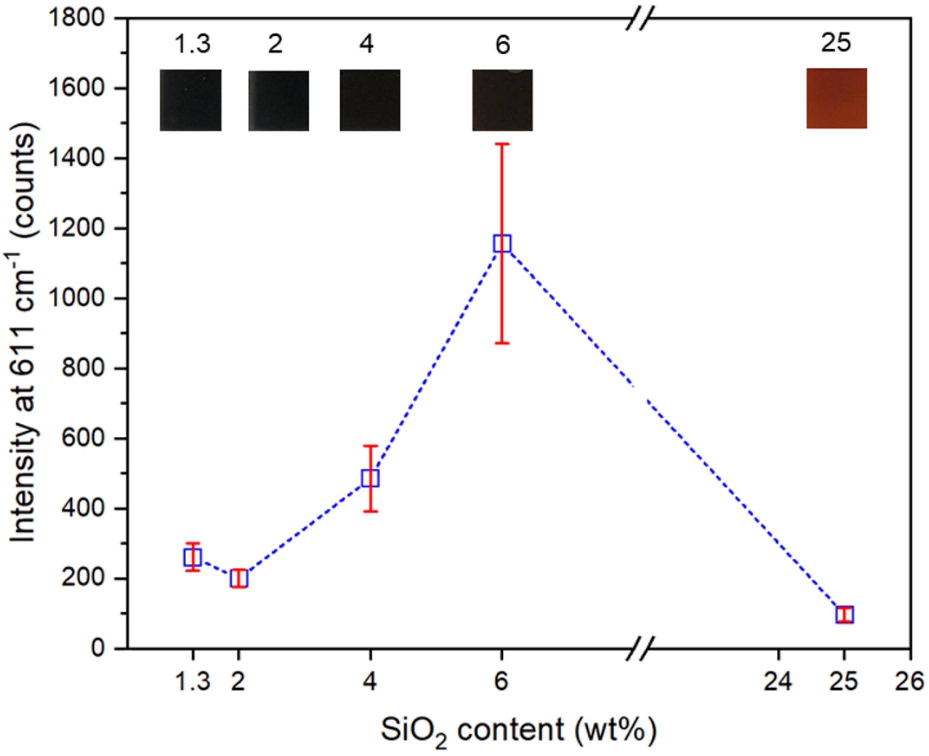

Figure S10. The intensity values at 611 cm^{-1} of SERS substrates using core-shell structured Ag-SiO_2 NPs with SiO_2 contents of 1.3 wt%, 2 wt%, 4 wt%, 6 wt%, and 25 wt%. The substrates were deposited on glass slides (76 mm X 52 mm X 1 mm) with a deposition time of 80s.[17] The inserted photos show part (27 mm X 27 mm, top view) of the deposited nanoparticle films. 2 µL of 10^{-4} M R6G solution was dropped on the substrate. After drying at room temperature, five different points at each substrate were detected to obtain the averaged value and the standard deviation. The SERS substrates containing Ag-SiO_2 NPs with the SiO_2 content of 6 wt% exhibits the highest sensitivity compared to other four groups, providing a rough range of SiO_2 content for optimizing the SERS performance. All data are represented mean ± SD. The sample size (n) is 5.

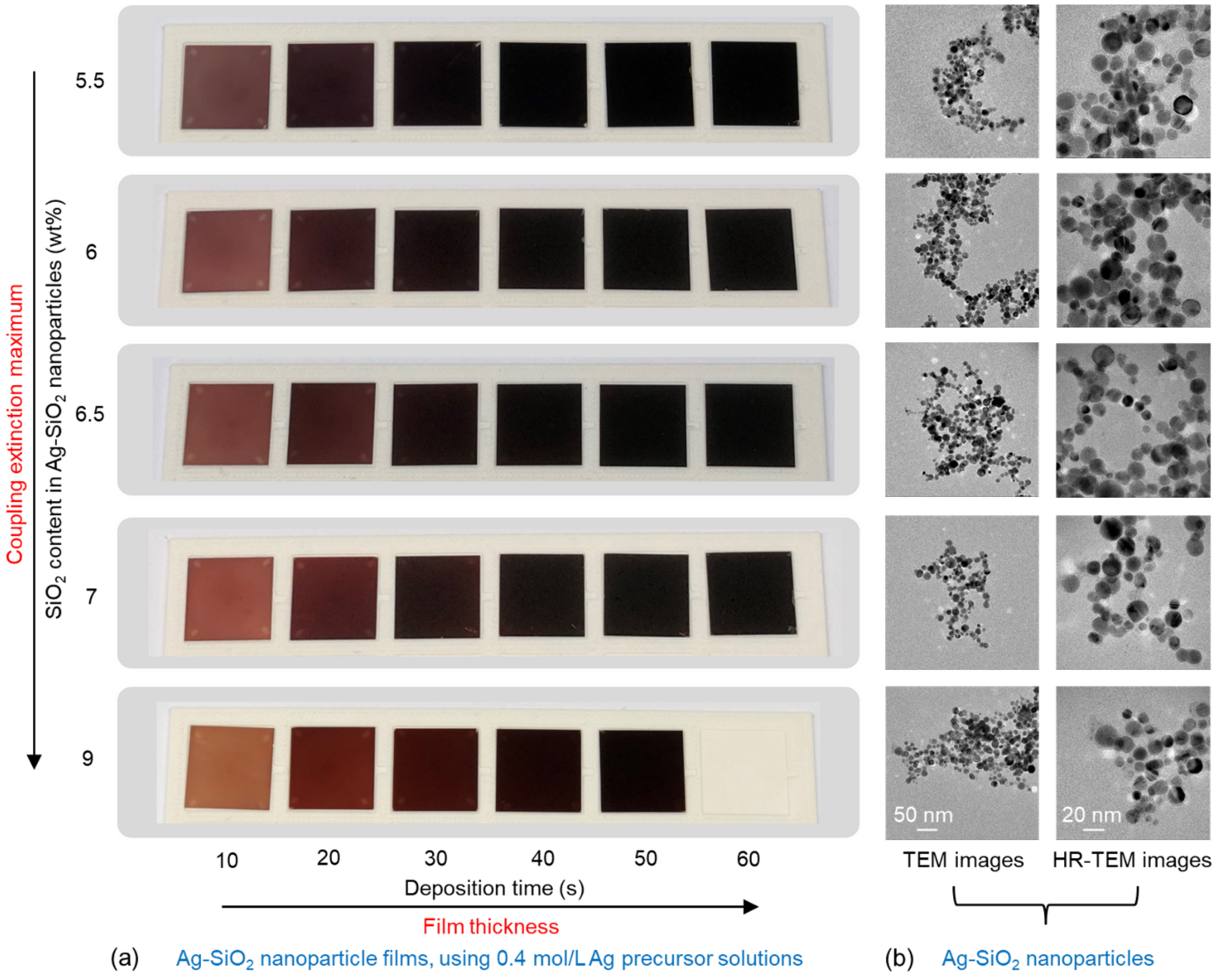

Figure S11. (a) Five groups of SERS substrates prepared using core-shell structured Ag-SiO_2 NPs with SiO_2 contents of 5.5 wt%, 6 wt%, 6.5 wt%, 7 wt%, and 9 wt%. During FSP deposition, 0.4 M Ag precursor solution was used. In each group of SERS substrates, the deposition time was increased to enhance the film thickness. (b) TEM images of core-shell structured Ag-SiO_2 NPs. The TEM images in each column have the same scale bar. (The particle size distributions are shown in Figure S2d-h).

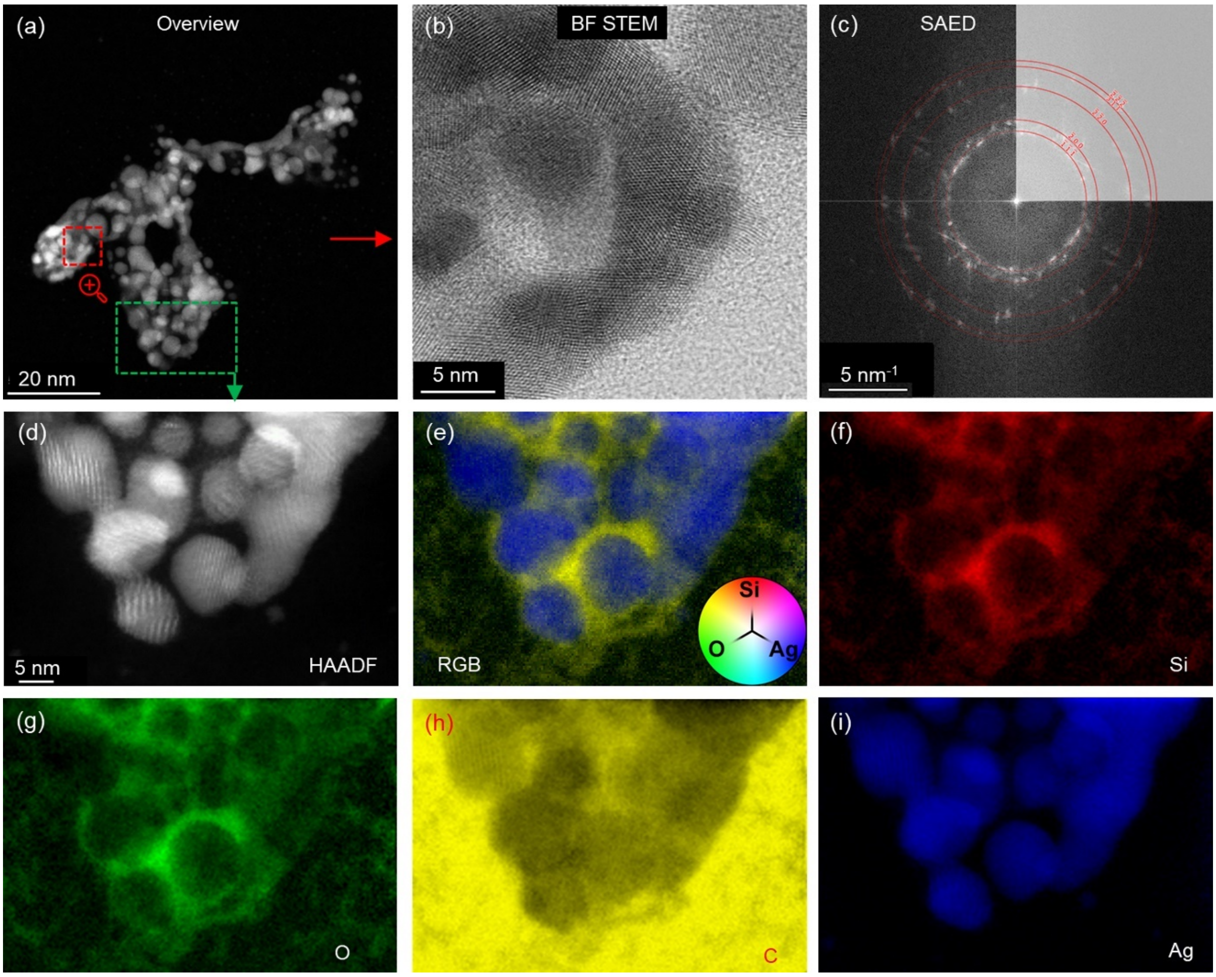

Figure S12. Ag-SiO_2 NPs prepared using 0.4 M Ag precursor solution and with the SiO_2 content of 6% in NPs. (a) STEM image showing the overview of fractal NPs. (b) High resolution BF STEM image of representative NPs and (c) their corresponding Selected Area Electron Diffraction (SAED) patterns. The diffraction rings in the SAED image fit well with the simulated results of Ag particles (red rings in the SAED image), indicating the presence of Ag NPs. (d) HAADF image of representative NPs. (e), (f), (g), (h), and (i) exhibit EDX/EELS fusion images with false colors showing the elemental compositions of silicon (f), oxygen (g), carbon (h), and silver (i). The Red-Green-Blue (RGB) color model was used in (e).

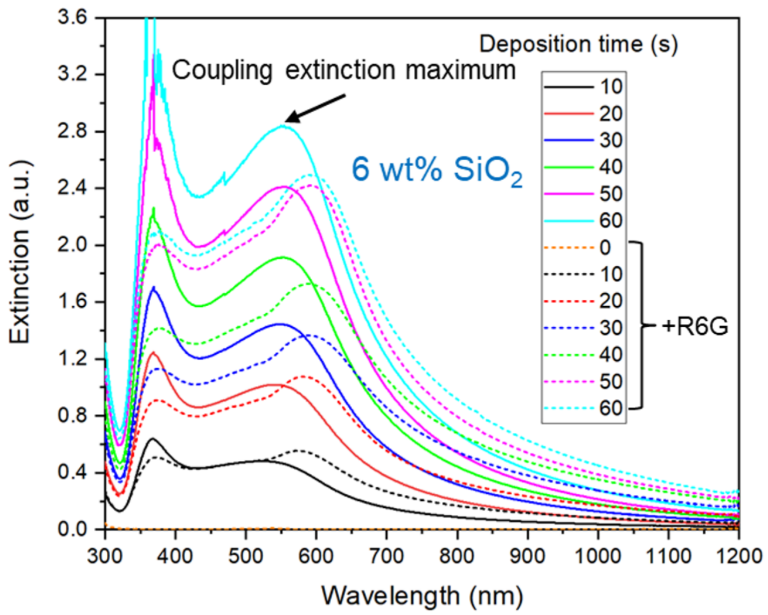

Figure S13. UV-Vis spectra of SERS substrates before (solid curves) and after (dashed curves) R6G deposition. The SERS substrates were deposited by core-shell structured Ag-SiO_2 NPs with SiO_2 contents of 6 wt%.

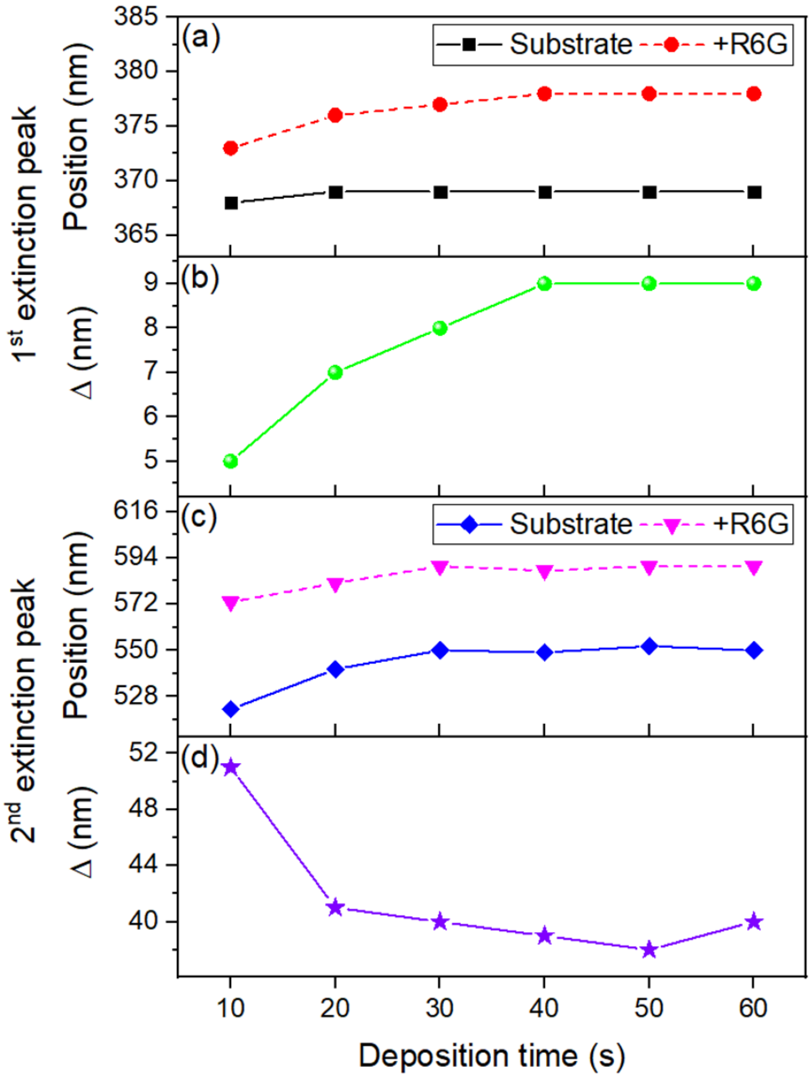

Figure S14. The first extinction peak positions before and after R6G deposition in UV-Vis spectra of SERS substrate (a) and their difference (b). The second extinction peak (LSPR λ_{max}) positions before and after R6G deposition in UV-Vis spectra of SERS substrate (c) and their difference (d). The SERS substrates were deposited by core-shell structured Ag-SiO_2 NPs with SiO_2 contents of 6 wt%. During FSP deposition, the 0.4 M Ag precursor solution was used.

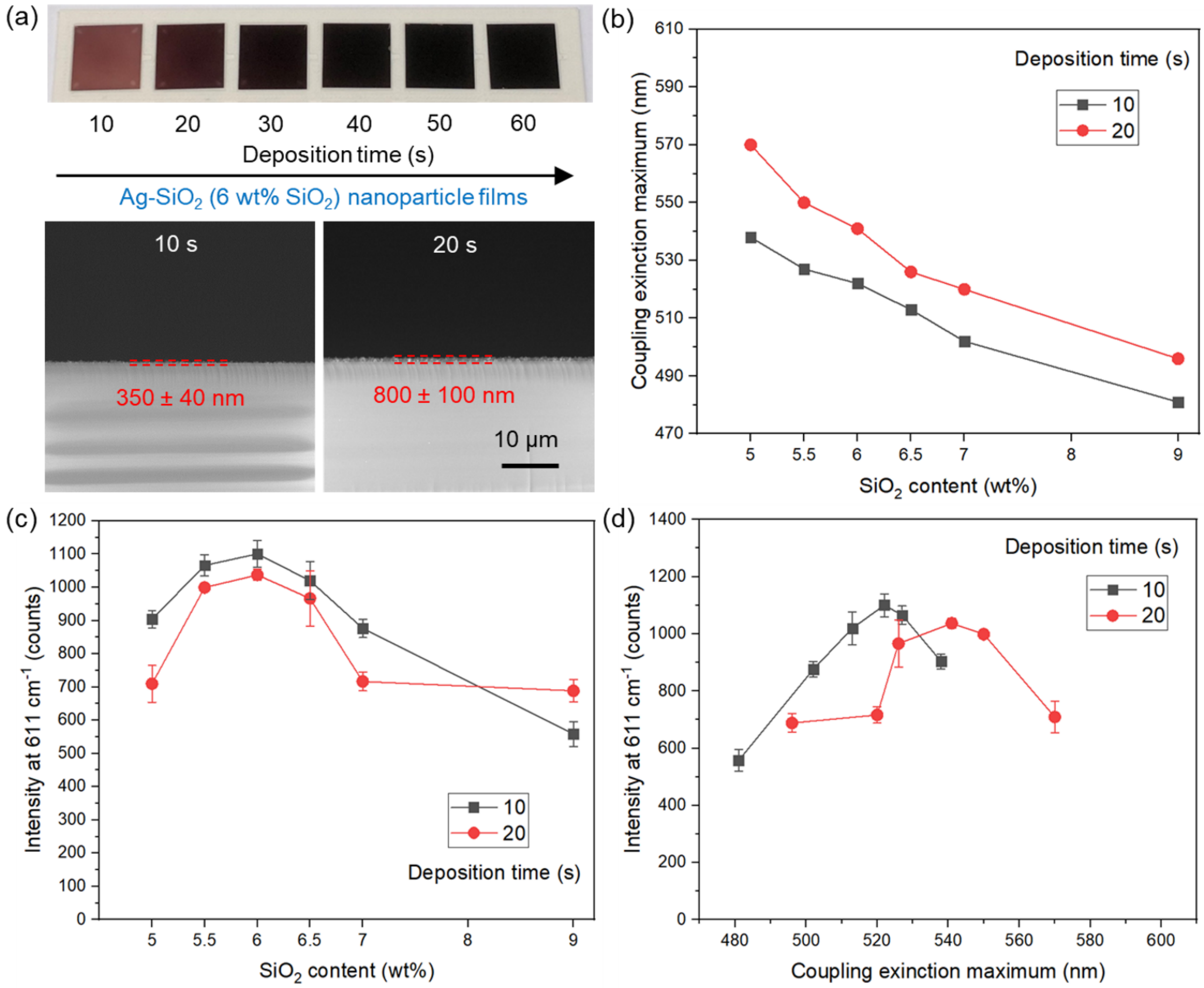

Figure S15. (a) The photo showing SERS substrates composing of core-shell structured Ag-SiO_2 NPs with SiO_2 contents of 6 wt%. The deposition times of 10 s, 20 s, 30 s, 40 s, 50 s, and 60 s were used to increase the film thickness. During FSP deposition, 0.4 M Ag precursor solution was used. The two SEM images (side view) of the substrates with deposition times of 10s and 20s were shown to indicate film thicknesses. (b) Coupling extinction maxima of two groups of SERS substrates with deposition times of 10 s and 20 s. These two groups of SERS substrates compose of core-shell structured Ag-SiO_2 NPs with SiO_2 contents of 5 wt%, 5.5 wt%, 6 wt%, 6.5 wt%, 7 wt%, and 9 wt%. During FSP deposition, 0.4 M Ag precursor solution was used. (c) The intensity values of 611 cm^{-1} peaks and their averaged values as a function of SiO_2 contents in Ag-SiO_2 NPs. (d) The intensity values of 611 cm^{-1} peaks and their averaged values as a function of coupling extinction maxima. All data are represented mean ± SD. The sample size (n) is 4-6.

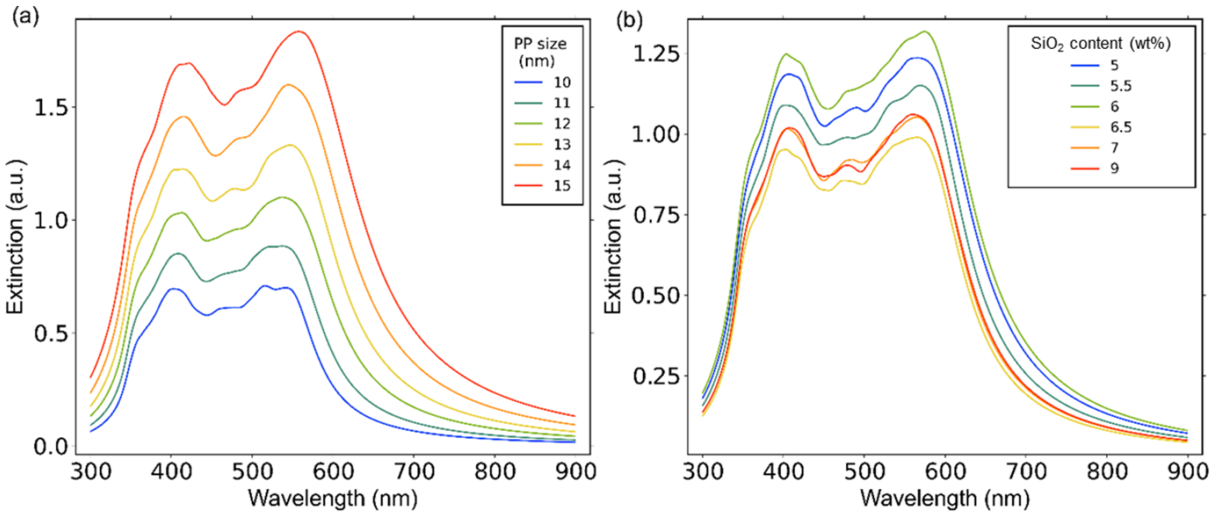

Figure S16. Simulation of extinction spectra of fractal like aggregates with (a) varying primary particle size and (b) with the primary particles sizes and geometric standard deviations corresponding to those measured from TEM images of aggregates synthesized with a given silica weight percentage (shown in Figure S2). PP in (a) represents primary particle.

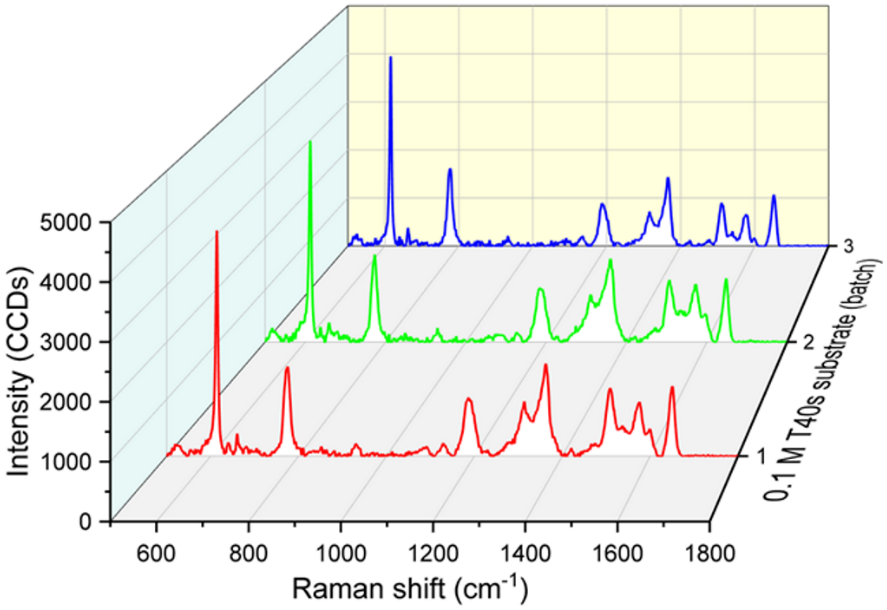

Figure S17. The SERS tests on three batches using a confocal Raman microscopy. The SERS substrates were prepared using 0.1 M Ag precursor solution, 5 wt% SiO_2 and the deposition time t_d = 40 s. These Raman spectra are averaged values from 6 different measurement points from the substrate. The averaged intensities of the detected points at 612 cm^{-1} are 3955 ± 916 CCD cts, 3951 ± 625 CCD cts, and 3868 ± 916 CCD cts, indicating good reproducibility of the fabrication process. All data are represented mean ± SD. The sample size (n) is 6.

Section S6. Rapid detection of pesticide residues on fruit surface.

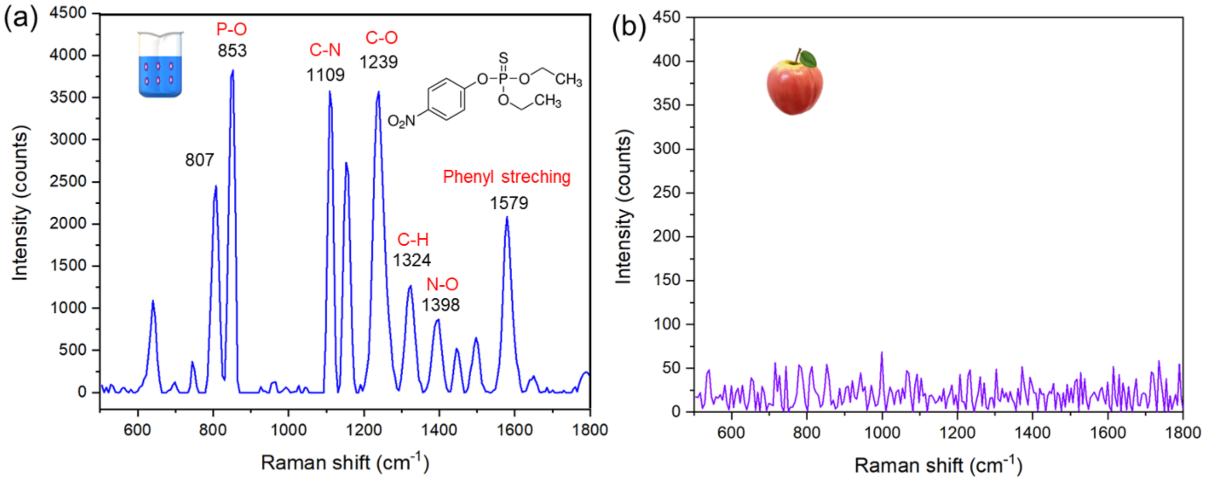

Figure S18. (a) The Raman spectra of 100 ppm parathion-ethyl in ethanol and its vibrational descriptions. The insert image shows the molecule structure of parathion-ethyl. The SERS spectra of parathion-ethyl exhibit peaks at 853 cm^{-1}, 1109 cm^{-1}, 1239 cm^{-1}, 1324 cm^{-1}, 1398 cm^{-1}, and 1579 cm^{-1}, which are assigned to the vibrations of P-O stretching, C-N stretching, C-O stretching, C-H bending, N-O stretching and phenyl stretching, respectively.[18] (b) The reference experiment for the rapid detection of pesticide residues on apple surface. During this reference experiment, pure ethanol instead of parathion-ethyl in ethanol was used and the rest operation process was the same as the pesticide experiment. The SERS spectra were the averaged value of ten detection points at the substrate surface. The intensity at 853 cm^{-1} is 54 counts with the standard deviation of 34 counts. It is in the range of the detection noise of the Raman spectrometer system. All data are represented mean ± SD. The sample size (n) is 10.

Section S7. The hypermodal data fusion method for EDX/EELS mapping.

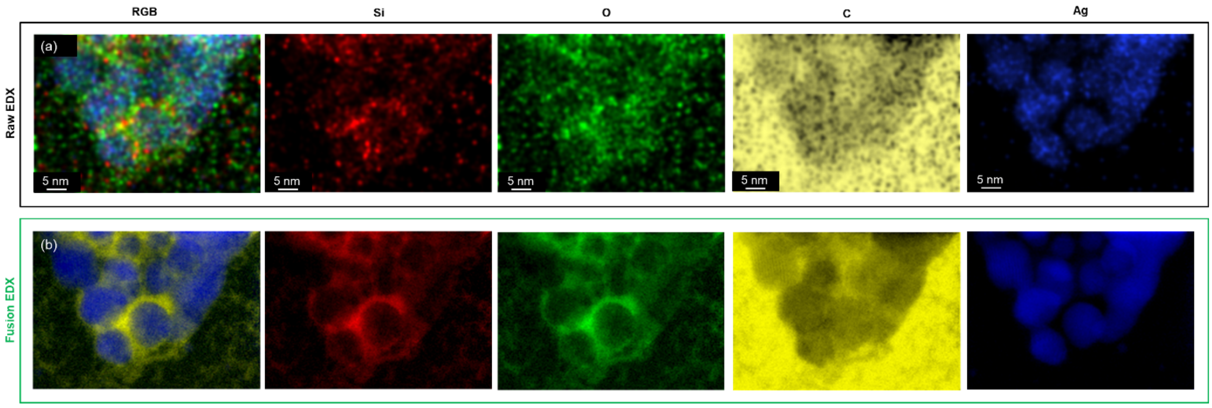

Figure S19. One representative example showcasing the benefits of the hypermodal data diffusion method for EDX/EELS mapping. (a) Raw EDX (after applying a spatial spectral blur to estimate missing values). (b) EDX after data fusion. Ag-SiO_2 NPs were prepared using 0.4 M Ag precursor solution and with the SiO_2 content of 6 wt% in NPs. The second, third, fourth, and fifth column exhibit images with false colors showing the elemental compositions of silicon, oxygen, carbon, and silver. The Red-Green-Blue (RGB) color model was used in first column. Note that, although the color yellow was used for the Carbon maps in column 4, this element was not included in the RGB composite image: the yellow here originates from mixing between Si (red) and O (green). The EDX mapping images have the same scale bar.

References

[1] B. N. Khlebtsov, V. A. Khanadeev, E. v. Panfilova, D. N. Bratashov, N. G. Khlebtsov, *ACS Applied Materials and Interfaces* **2015**, *7*, 6518.

[2] T. Itoh, Y. S. Yamamoto, *The Journal of Chemical Physics* **2018**, *149*, 244701.

[3] J. Zhu, L. Sun, Y. Shan, Y. Zhi, J. Chen, B. Dou, W. Su, *Vacuum* **2021**, *187*.

[4] J. Ju, W. Liu, C. M. Perlaki, K. Chen, C. Feng, Q. Liu, *Scientific Reports* **2017**, *7*, 1.

[5] N. Dar, K. Y. Chen, Y. T. Nien, N. Perkas, A. Gedanken, I. G. Chen, *Applied Surface Science* **2015**, *331*, 219.

[6] J. Fang, Y. Yi, B. Ding, X. Song, *Applied Physics Letters* **2008**, *92*, 1.

[7] V. S. Vendamani, S. V. S. N. Rao, A. P. Pathak, V. R. Soma, *RSC Advances* **2020**, *10*, 44747.

[8] M. Zhang, A. Zhao, H. Sun, H. Guo, D. Wang, D. Li, Z. Gan, W. Tao, *Journal of Materials Chemistry* **2011**, *21*, 18817.

[9] J. Wang, Y. Zhao, H. Huang, T. Cong, H. Zhang, K. Liu, L. Pan, *Applied Surface Science* **2020**, *527*, 146948.

[10] J. Liu, P. peng Jiang, C. He, X. hong Jiang, L. de Lu, *Journal of Materials Science* **2018**, *53*, 3443.

[11] C. Li, C. Yang, S. Xu, C. Zhang, Z. Li, X. Liu, S. Jiang, Y. Huo, A. Liu, B. Man, *Journal of Alloys and Compounds* **2017**, *695*, 1677.

[12] Y. Q. Wang, S. Ma, Q. Q. Yang, X. J. Li, *Applied Surface Science* **2012**, *258*, 5881.

[13] C. Zhang, S. Chen, Z. Jiang, Z. Shi, J. Wang, L. Du, *ACS Applied Materials & Interfaces* **2021**, *13*, 29222.

[14] D. Xu, F. Teng, Z. Wang, N. Lu, *ACS Applied Materials and Interfaces* **2017**, *9*, 21548.

[15] B. Yuan, J. Guo, S. Bai, *Journal of Materials Chemistry C* **2020**, *8*, 6478.

[16] D. Ge, J. Wei, J. Ding, J. Zhang, C. Ma, M. Wang, L. Zhang, S. Zhu, *ACS Applied Nano Materials* **2020**.

[17] P. Merkl, S. Zhou, A. Zaganiaris, M. Shahata, A. Eleftheraki, T. Thersleff, G. A. Sotiriou, *ACS Applied Nano Materials* **2021**, *4*, 5330.

[18] D. Lee, S. Lee, G. H. Seong, J. Choo, E. K. Lee, D. G. Gweon, S. Lee, *Applied Spectroscopy* **2006**, *60*, 373.
